# A machine learning model for in-hospital mortality prediction in patients with sepsis-related myocardial injury

**DOI:** 10.1371/journal.pdig.0001758

**Published:** 2026-10-12

**Authors:** Da-cheng Wang, Yan-pin Liu, Yu-ting Wang, Xiao-huan Zhuang, Xiao-peng Yin, Xin-yuan Zhang, Yan Zhuang

**Affiliations:** 1 Department of Intensive Care Unit, Affiliated Hospital of Nanjing University of Chinese Medicine, Nanjing, Jiangsu, China; 2 The First Clinical Medical College, Nanjing University of Chinese Medicine, Nanjing, Jiangsu, China; National Yang Ming Chiao Tung University, TAIWAN

## Abstract

Sepsis-related myocardial injury (SRMI) is associated with high mortality, but the transportability of prediction models across heterogeneous cohorts remains uncertain. We developed and externally validated a machine learning model for in-hospital mortality prediction in SRMI and evaluated its discrimination, calibration, interpretability, and distributional shift across cohorts. Using MIMIC-IV for development and MIMIC-III and a local cohort for external validation (n = 1,226, 962, and 280, respectively), we compared eight classification algorithms. A two-stage feature-selection strategy, recursive feature accumulation and sequential elimination with incremental feature evaluation, ranked 31 candidate variables to derive a parsimonious predictor set. The selected model was evaluated for discrimination, precision-recall performance, calibration, clinical utility, and risk stratification. Logistic recalibration, population stability index (PSI) analysis, and SHapley Additive exPlanations (SHAP) were used to assess probability alignment, cross-cohort distributional shift, and model interpretability. Feature selection reduced 31 candidate variables to 8 predictors with little loss of cross-validated performance (AUC, 0.819 with 31 variables versus 0.810 with 8 variables). The selected random forest model achieved an AUC of 0.819 (bootstrap 95% CI, 0.787-0.848) and a PR-AUC of 0.702 (0.657-0.751) in MIMIC-III, and an AUC of 0.758 (0.693-0.816) and a PR-AUC of 0.525 (0.435-0.630) in the local cohort. Recalibration reduced the Brier score from 0.162 to 0.147 in MIMIC-III and from 0.233 to 0.196 in the local cohort. PSI indicated minimal distributional shift between MIMIC-IV and MIMIC-III but substantial shift in the local cohort, particularly for SOFA score, respiratory rate, and prothrombin time. SHAP identified prothrombin time, invasive ventilation, lactate, temperature, and urine output as major contributors to model predictions. In two external cohorts, an 8-variable random forest model showed moderate discrimination for mortality prediction in SRMI. The evaluation of external performance, calibration, distributional shift, and SHAP-based explanations provides an assessment of model transportability. Prospective validation is needed before clinical deployment.

## Author summary

Sepsis can damage the heart and is associated with a high risk of death in intensive care. Clinicians need practical ways to identify patients with sepsis-related myocardial injury who may need closer monitoring after the first day in the intensive care unit. We developed a prediction model using routinely collected information from the first 24 hours of ICU care, including age, illness severity, breathing support, urine output, vital signs, lactate, and prothrombin time. We developed the model in one large critical-care database and tested it in a second database and an independent local hospital cohort. The model showed moderate ability to distinguish survivors from non-survivors in both external cohorts. We also assessed whether its predicted risks were well calibrated, how patient characteristics differed between hospitals, and which variables contributed most to predictions. The model is intended to support early risk stratification, not to replace clinical judgment or established severity scores. Because the local cohort was small and the study was retrospective, prospective multicenter validation and local assessment are needed before clinical use.

## 1 Introduction

Sepsis is a life-threatening organ dysfunction caused by a dysregulated host response to infection, and it remains a major global public health challenge [1,2]. Recent epidemiological estimates indicate that approximately 49 million cases of sepsis occur annually worldwide, resulting in more than 11 million deaths that account for nearly 20% of all-cause mortality [2,3]. Among the organ systems affected during sepsis, the cardiovascular system is particularly vulnerable, and cardiac involvement is closely associated with adverse clinical outcomes [4,5].

Sepsis-related myocardial injury (SRMI) is a common and severe cardiovascular complication in critically ill patients with sepsis [4–7] that is typically characterized by elevated cardiac biomarkers, including cardiac troponin and N-terminal pro-brain natriuretic peptide, with or without concomitant cardiac dysfunction [5–8]. Previous studies have shown that myocardial injury during sepsis is associated with substantially increased short-term and long-term mortality [6,7,9]. Mechanistically, SRMI is considered to arise from a complex interplay of systemic inflammation, microcirculatory dysfunction, oxidative stress, mitochondrial injury, and metabolic derangement, which together impair myocardial energy utilization and contractile performance [5,10–13].

Despite its clinical relevance, early risk stratification in SRMI remains challenging. Conventional severity scores, such as the Sequential Organ Failure Assessment (SOFA) score and Acute Physiology and Chronic Health Evaluation II (APACHE II), are not specifically designed to capture cardiac-specific injury patterns and may inadequately reflect the prognostic heterogeneity of this population [14,15]. Similarly, individual biomarkers are useful for identifying myocardial injury but have limited ability to provide robust individualized mortality prediction when used in isolation [8,16,17]. These limitations underscore the need for more effective prediction approaches capable of integrating multidimensional clinical information.

Machine learning (ML) has emerged as a promising approach for risk prediction in critical care because it can model complex nonlinear associations and interactions between clinical variables [18,19]. Previous studies have shown that ML-based models can achieve favorable performance for mortality prediction in sepsis and septic shock [19–21]. However, many published models remain limited by insufficient external validation, performance degradation across heterogeneous cohorts, and suboptimal calibration, all of which restrict real-world transportability and clinical utility [22–25].

To address these issues, we sought to develop and externally validate an ML-based prediction model for in-hospital mortality in patients with SRMI using two large critical care databases, MIMIC-IV and MIMIC-III, together with a real-world local cohort [26,27]. A two-stage feature selection framework, a random forest-based ranking approach followed by sequential elimination with incremental feature evaluation (RFA-SEIFE), was used to derive a parsimonious set of clinically relevant predictors [28,29]. Model interpretability was further enhanced using SHapley Additive exPlanations (SHAP) analysis [23,30]. In addition, strict recalibration was performed in the external validation cohorts to improve probability estimation, and population stability index (PSI) analysis was used as a supplementary assessment of cross-cohort distributional shift [24,25]. Our goal with this multicohort design was to evaluate the transportability of an early risk-stratification model and to identify requirements for local calibration or updating before clinical use.

## 2 Methods

### 2.1 Ethics statement

This study was conducted using data from the publicly available, de-identified MIMIC-III and MIMIC-IV databases, as well as a local retrospective cohort from the Affiliated Hospital of Nanjing University of Chinese Medicine. Access to the MIMIC databases was obtained after completion of the required Collaborative Institutional Training Initiative (CITI) training (Certificate ID: 55981932). The use of MIMIC-III and MIMIC-IV complied with the data-use agreements approved by the Institutional Review Boards of the Massachusetts Institute of Technology and Beth Israel Deaconess Medical Center, and the requirement for individual informed consent was waived because all data were de-identified. The local cohort study was approved by the Ethics Committee of the Affiliated Hospital of Nanjing University of Chinese Medicine, and the requirement for informed consent was waived due to the retrospective design and use of de-identified clinical data (Ethics approval number: SJCX25_1001).

This study was reported in accordance with the TRIPOD-AI recommendations for prediction model development and validation. The completed reporting checklist is provided in S1 Checklist TRIPOD AI Checklist.

### 2.2 Study population and data sources

This multicohort retrospective study was conducted using three independent datasets, including a development cohort derived from the Medical Information Mart for Intensive Care IV (MIMIC-IV) database, an external validation cohort from the MIMIC-III database, and a real-world local cohort from the intensive care unit (ICU) of the Affiliated Hospital of Nanjing University of Chinese Medicine. The MIMIC-IV (version 2.2) and MIMIC-III (version 1.4) databases are large, publicly available critical care databases that contain de-identified health-related data from patients admitted to the Beth Israel Deaconess Medical Center [26,27]. The local cohort consisted of consecutive patients admitted to the ICU between January, 2022 and January, 2026. Clinical data were extracted from the hospital electronic medical record system and ICU database, and all patient information was anonymized prior to analysis.

Sepsis and septic shock were defined according to the Sepsis-3 criteria. Sepsis was identified as suspected or confirmed infection accompanied by an increase in Sequential Organ Failure Assessment (SOFA) score of ≥2 points from baseline, and septic shock was defined as persistent hypotension requiring vasopressor therapy to maintain a mean arterial pressure ≥65 mmHg and a serum lactate level >2 mmol/L despite adequate fluid resuscitation. The clinical SRMI concept was informed by the 2025 expert consensus [31]; however, the reproducible operational definition was prespecified separately for each cohort (S1 Table). In MIMIC-IV, eligible patients had Sepsis-3 within the first ICU day and at least one elevated troponin measurement recorded both within the first 24 h after ICU admission and within ±24 h of the operational sepsis time. Troponin T (itemid 51003), troponin I (51002), and high-sensitivity troponin I (52642) were retrieved from labevents. An elevated result was defined as a value above its row-level ref_range_upper when that upper limit was available and positive; otherwise, the laboratory abnormal flag was used. Thus, the consensus 99th-percentile concept was not retrospectively imposed as one common numeric cutoff in MIMIC-IV. In MIMIC-III, troponin I (51002) and troponin T (51003) were retrieved over the same time windows. Because a stable row-level upper reference limit was not available, elevation was defined by the laboratory abnormal flag; no universal fixed numeric cutoff was applied.

For the MIMIC cohorts, ACS and competing causes were excluded using admission-level diagnosis and procedure codes rather than chart review (S1 Table). The primary exclusion algorithm removed acute myocardial infarction/ACS, pulmonary embolism, myocarditis, cardiac arrest, major cardiac surgery or intervention, CPR, and severe chest trauma. In MIMIC-IV, a qualifying diagnosis or procedure recorded at any time during the hospitalization was used as a conservative proxy because the extraction did not support reliable temporal ordering of all procedure records relative to troponin elevation. Patients also had to be aged ≥18 years, have Sepsis-3 and the cohort-specific SRMI criterion, be in their first ICU stay, and have the core data required to establish the phenotype.

In the local validation cohort, cardiac troponin I was measured using the Beckman Coulter Access AccuTnI + 3 chemiluminescent immunoassay. Troponin elevation was defined as at least one conventional cTnI concentration >40 pg/mL within the first 24 h after ICU admission. Because the Access AccuTnI + 3 assay is distinct from the Beckman Coulter Access high-sensitivity troponin I assay, the biomarker was reported as conventional cTnI rather than high-sensitivity cTnI. The first-24-h maxima of CK-MB and BNP/NT-proBNP were retained for clinical characterization but did not determine SRMI eligibility. ACS and competing causes were excluded by manual review of the medical record.

Troponin was not included among the candidate predictors because it was used to establish SRMI eligibility. Using an assay-dependent troponin concentration as a predictor after enrolment on troponin elevation would conflate phenotype ascertainment with prognostic modelling and would not provide a common measurement scale across the three assay environments. Accordingly, troponin and the locally available CK-MB and BNP/NT-proBNP measurements were used for cohort characterization rather than as cross-cohort candidate predictors.

### 2.3 Machine-learning framework

The modelling strategy was designed to develop and externally validate an early prediction model for in-hospital mortality in SRMI. Candidate predictors were obtained from the first 24 hours after ICU admission. The workflow comprised predictor preprocessing, two-stage feature selection, comparison of eight candidate classification algorithms, internal validation in the development cohort, and external evaluation in the MIMIC-III and local cohorts. Recalibration, PSI analysis, and SHAP analysis were subsequently used to assess probability alignment, distributional shift, and model interpretability, respectively.

All candidate predictors were restricted to observations recorded from ICU admission until 24 hours after ICU admission. The model was therefore intended for risk stratification after completion of the first ICU day, rather than at the instant of ICU admission. The aggregation rule was prespecified by variable type: SOFA was the first-24-hour derived score; lactate and laboratory severity markers used the maximum recorded value, except hemoglobin and platelet count, which used the minimum recorded value; heart rate, mean blood pressure, respiratory rate, and temperature used the mean recorded value; urine output was cumulative over the first 24 hours; and invasive ventilation and vasopressor use were coded as any exposure during the first 24 hours. Age, sex, and pre-existing comorbidities were defined at admission or from pre-existing diagnoses. No measurements or treatments recorded after the 24-hour predictor window were used for model development or validation. Invasive ventilation and vasopressor use may reflect both illness severity and early management and are interpreted as prediction variables, not causal effects. Full variable-level definitions are provided in S2 Table.

The analysis focused on how established algorithms were implemented and evaluated in this study rather than on general descriptions of their underlying principles. After the dataset-level KNN imputation described below, preprocessing used during feature selection and model fitting was fitted using development data only, with fold-specific fitting during cross-validation to limit information leakage.

### 2.4 Predictor processing and feature selection

Patients with >30% overall missingness across candidate predictors were excluded. Candidate variables with >30% missingness were then removed. Residual missing values were imputed using k-nearest neighbours (k = 10), yielding complete analytic datasets for subsequent model development. For feature selection, continuous predictors were winsorized at the 1st and 99th percentiles using development-data thresholds and standardized, and categorical predictors were one-hot encoded. A two-stage feature-selection strategy was then used to derive a parsimonious predictor set: random-forest-based ranking approach (RFA), followed by sequential elimination with incremental feature evaluation (SEIFE) [28,29]. In the RFA stage, permutation importance was aggregated to the original variable level for one-hot encoded predictors and used to rank candidate variables.

Variables were incrementally added according to this ranking. At each step, repeated stratified cross-validation was used to estimate AUC and PR-AUC, and the best-performing subset was carried forward.

In the SEIFE stage, variables were iteratively considered for removal in ascending order of importance. A variable was removed only when its exclusion did not reduce AUC beyond the predefined tolerance; the process stopped when no further removal was acceptable or the prespecified minimum number of variables was reached.

RFA-SEIFE used repeated stratified 5-fold cross-validation with three repetitions to evaluate candidate subsets in the development cohort (AUC as the primary metric and PR-AUC as supportive). Preprocessing for each subset-performance evaluation was fitted on the corresponding training partition only. However, the initial ranking and the final eight-predictor set were derived using the full development cohort and then held fixed for subsequent model-performance evaluation; feature selection was not rerun within each outer performance-validation fold. This non-nested selection approach is acknowledged as a potential source of optimistic internal-performance estimation. The final predictor set was also reviewed for clinical plausibility and feasibility of routine collection.

### 2.5 Model development, tuning, and validation

Eight established classification algorithms were implemented: logistic regression, random forest, extreme gradient boosting (XGBoost), extra trees, support vector machine, k-nearest neighbors, multilayer perceptron, and AdaBoost. The models were compared using the same development, validation, and performance-evaluation framework.

For reproducibility, random_state was set to 42 throughout. Internal performance was assessed by repeated stratified 5-fold cross-validation with three repetitions. Hyperparameters for the eight final candidate algorithms were optimized by randomized search in the development cohort using stratified 5-fold cross-validation, ROC-AUC scoring, and 20 sampled parameter sets per algorithm; the full search spaces and selected configurations are reported in S3 Table. Missing values were imputed using k-nearest neighbours (k = 10) before model development. For subsequent model training, continuous variables were winsorized at the development-training 1st and 99th percentiles and standardized, and binary and categorical variables were one-hot encoded; these preprocessing steps were fitted using the relevant training observations only. As shown in S4 Table, variable-level missingness was below the prespecified 30% exclusion threshold; KNN imputation was used to retain multivariable structure while handling the remaining missing values. Class imbalance was addressed with class_weight = balanced for logistic regression, random forest, extra trees, and support vector machine; for XGBoost, scale_pos_weight was set to the negative-to-positive class ratio in the relevant development-training data; no explicit class weighting was used for k-nearest neighbours, multilayer perceptron, or AdaBoost. Because class weighting can alter the scale of predicted probabilities, we conducted a sensitivity analysis in which the final random forest was refitted without class weighting while retaining the same eight predictors, fixed hyperparameters, and development-derived preprocessing. Probability performance was compared before threshold selection using AUC, PR-AUC, Brier score, calibration intercept, and calibration slope. For threshold-dependent descriptive measures, a Youden-index threshold was selected from repeated development-cohort out-of-fold probabilities and applied unchanged to both external cohorts. Although the selected hyperparameters were then held fixed during repeated cross-validation, hyperparameter selection itself was not rerun within each outer performance-validation fold. Pre-imputation missingness by cohort is reported in S4 Table.

After tuning, internal performance was estimated with repeated stratified 5-fold cross-validation (three repetitions). In each fold, the complete pipeline was reconstructed and evaluated in the held-out fold. Performance measures included AUC, PR-AUC, Brier score, accuracy, balanced accuracy, F1 score, recall, and precision; internal results are reported as means and standard deviations across repetitions.

Following internal validation, each model was retrained using the full development cohort and evaluated in the two independent external cohorts. Development-derived preprocessing parameters were applied to external data to emulate deployment. The primary model was selected using the prespecified comparison of external discrimination, calibration, and robustness, then carried forward for recalibration, PSI, and SHAP analyses.

### 2.6 Recalibration

A post hoc recalibration procedure was performed to evaluate the calibration performance of the selected model under cross-cohort conditions. Given the known variability in baseline risk and predictor distributions across datasets, calibration of predicted probabilities is essential for reliable clinical interpretation. A logistic recalibration approach was applied based on the development cohort. Repeated stratified cross-validation in the development cohort was first used to generate out-of-fold predicted probabilities for every participant. A logistic calibration model was then fitted to the observed outcomes and these out-of-fold probabilities as follows:


$$\begin{array}{c} ogit(Padj)=\alpha +\beta \cdot logit(Ppred), \end{array}$$

where Ppred represents the original predicted probability from the model, Padj denotes the recalibrated probability, and α and β correspond to the calibration intercept and slope, respectively.

Importantly, the recalibration mapping was fitted to repeated stratified 5-fold out-of-fold probabilities in the development cohort (three repetitions; n = 1,226), rather than to resubstitution predictions from the final random forest. The fitted mapping was logit(Padj) = -0.414 + 1.156 x logit(Ppred). It was then applied unchanged to probabilities from the final development model and to the external validation cohorts (MIMIC-III and the local cohort) without refitting. This design assessed transportability under a fixed development-derived probability adjustment rather than optimal cohort-specific calibration or local model updating.

The effectiveness of recalibration was assessed using multiple metrics, including the Brier score, calibration intercept, and calibration slope. Calibration curves were also constructed to visually compare predicted probabilities with observed event rates across risk strata. This recalibration strategy does not account for cohort-specific baseline risk differences and therefore may not fully eliminate miscalibration in external datasets. Instead, it provides a conservative assessment of whether the model retains usable probability structure when applied to new populations without additional parameter updating.

### 2.7 Benchmark comparison with SOFA

To contextualize performance against an established severity score, we compared the selected random forest model with SOFA in all three cohorts. In the development cohort, random-forest performance was assessed using stored out-of-fold probabilities and SOFA performance using five-fold stratified out-of-fold probabilities from a logistic calibration model. This logistic mapping was fitted once in the development cohort and then applied unchanged to the MIMIC-III and local cohorts. AUC, PR-AUC, and Brier score were reported for both approaches. Paired AUC differences were tested using the DeLong method. Stratified nonparametric bootstrap resampling (2,000 replicates; seed 20260803) provided percentile 95% confidence intervals for performance and incremental-value measures. Continuous (category-free) net reclassification improvement (NRI) and integrated discrimination improvement (IDI) were calculated for random forest relative to SOFA; their two-sided bootstrap P values were derived from the empirical distribution. APACHE II was not compared because a harmonized APACHE II score, or the complete component data needed to reconstruct it reproducibly, was not available across all three analytic cohorts.

### 2.8 Model Interpretability using SHAP

Model interpretability was assessed using the SHAP method, which quantifies the contribution of each feature to individual predictions based on cooperative game theory [30]. Since recalibration only modifies the output probabilities without altering the internal structure of the model, this analysis was conducted on the final model prior to recalibration. Specifically, the primary SHAP analysis was performed in the MIMIC-III cohort to characterize feature-contribution patterns, with the local cohort used for supplementary assessment.

Three levels of interpretability were evaluated. 1. Global feature importance: Mean absolute SHAP values were calculated to rank feature importance, and summary bar plots were generated to identify the most influential predictors. 2. Feature effect and distribution patterns: SHAP beeswarm plots were used to visualize the distribution and directionality of feature effects across individuals. Dependence plots were further constructed for key variables to explore nonlinear relationships and potential interaction effects. 3. Individual prediction explanation: Representative high-risk and low-risk cases were selected to generate SHAP waterfall plots, illustrating the contribution of each feature to individual predictions. SHAP decision plots were also used to depict cumulative prediction pathways across multiple patients. This approach reflects a transportability-oriented recalibration rather than cohort-specific recalibration and should thus be interpreted accordingly.

### 2.9 Sensitivity analyses: Distribution shift, harmonization, and class weighting

To assess the impact of distributional differences across datasets, we used the population stability index (PSI) to quantify feature distribution shifts between the development cohort and external validation cohorts [24]. For continuous variables, binning was performed based on deciles derived from the development cohort, and the same bin boundaries were applied to external datasets. PSI was calculated as:


$$\begin{array}{c} PSI=\sum \left(p_{i}-q_{i}\right)\text{ln}\left(\frac{p_{i}}{q_{i}}\right), \end{array}$$

where $p_{i}$ and $q_{i}$ represent the proportions of observations in each bin for the development and external cohorts, respectively. For categorical variables, PSI was computed based on category frequencies and interpreted as follows: < 0.10 (stable), 0.10-0.25 (moderate shift), and >0.25 (substantial shift). For variables with high PSI values, a development cohort-anchored harmonization strategy was applied. Specifically, binning thresholds derived from the development cohort were used to transform corresponding variables in external cohorts, aiming to improve cross-cohort comparability while preserving relative ordering.

The PSI interpretation bands were used as descriptive screening conventions rather than inferential cut points. Variables with PSI > 0.25 were examined in the development-anchored harmonization sensitivity analysis; this procedure did not alter primary model training or the primary performance estimates.

To evaluate the impact of distribution shift and harmonization, model performance (AUC, PR-AUC, and Brier score) was compared before and after harmonization. This harmonization was a sensitivity analysis only and was not incorporated into the final model. All primary results were based on the original variables to ensure interpretability and reproducibility.

Separately, we assessed the impact of class weighting on the selected random forest. The published eight predictors, fixed random-forest hyperparameters, development cohort, and development-derived preprocessing were retained, and the model was refitted with class_weight = None rather than class_weight = balanced. Threshold-independent performance was compared before recalibration and before threshold selection using AUC, PR-AUC, Brier score, calibration intercept, and calibration slope. For descriptive threshold-dependent measures, the Youden index was selected from repeated stratified 5-fold development-cohort out-of-fold probabilities (three repetitions) and the resulting threshold was applied unchanged to the external cohorts. This post hoc sensitivity analysis was not used to reselect the primary model.

### 2.10 Statistical analysis

All statistical analysis was performed using Python 3.9 (Python Software Foundation, Wilmington, DE, USA), primarily with the pandas, NumPy, scikit-learn, SciPy, and matplotlib libraries. Prior to analysis continuous variables were expressed as mean ± standard deviation (SD) or median with interquartile range (IQR), depending on data distribution, and normality was assessed using the Shapiro-Wilk test. For comparisons between the three cohorts (MIMIC-IV, MIMIC-III, and the local cohort), one-way analysis of variance (ANOVA) was used for normally distributed variables, and the Kruskal-Wallis test was used for nonnormally distributed variables. Categorical variables were expressed as counts and percentages and compared using the chi-squared test or Fisher’s exact test, as appropriate.

Model performance was evaluated using discrimination, calibration, and classification metrics. Specifically, discrimination was assessed using the area under the receiver operating characteristic curve (AUC) and the area under the precision-recall curve (PR-AUC). Calibration was evaluated using the Brier score and calibration curves. Decision curve analysis (DCA) was conducted to assess clinical utility. Population stability index (PSI) was used to quantify distributional differences between the development and external validation cohorts, and recalibration performance was assessed using calibration intercept, calibration slope, and Brier score. All statistical tests were two-sided, and a P value < 0.05 was considered to indicate a statistically significant test result.

## 3 Results

### 3.1 Baseline characteristics of the study population

A total of 2,468 patients with SRMI were included across the three cohorts, comprising 1,226 patients in the development cohort (MIMIC-IV), 962 patients in the external validation cohort (MIMIC-III), and 280 patients in the local cohort. The detailed patient selection process is illustrated in Fig 1. Significant differences in baseline characteristics were observed among the three cohorts (Table 1). In particular, patients in the local cohort were older compared to those in the MIMIC-IV and MIMIC-III cohorts (69.30 ± 16.31 vs. 62.00 ± 17.02 and 60.73 ± 15.92 years, respectively; P < 0.001). In terms of disease severity, both external validation cohorts exhibited higher SOFA scores than the development cohort (median: 8.00 and 7.00 vs. 4.00, P < 0.001). Similarly, GCS scores were significantly lower in the local cohort, indicating more severe neurological impairment. All patients met the cohort-specific SRMI troponin-elevation criterion. Assay-specific first-24-h cardiac biomarker distributions are reported descriptively in Table 1; these measurements were not pooled or compared across cohorts because the assays, units, and reference-limit conventions differed.

**Table 1:** Baseline characteristics of the development and external validation cohorts.

Variable	MIMIC-IV (n = 1226)	MIMIC-III (n = 962)	Local (n = 280)	P value
**Demographics**				
Sex (male), n (%)	644 (52.53)	575 (59.77)	177 (63.21)	<0.001
Age (years), Mean ± SD	62.00 ± 17.02	60.73 ± 15.92	69.30 ± 16.31	<0.001
**Severity scores**				
SOFA score, Median (IQR)	4.00 (3.00–6.00)	7.00 (5.00–10.00)	8.00 (5.00–12.00)	<0.001
GCS score, Median (IQR)	15.00 (12.00–15.00)	15.00 (13.00–15.00)	10.50 (7.00–15.00)	<0.001
**Cardiac biomarkers (assay-specific)**				
Patients meeting cohort-specific SRMI troponin criterion, n (%)	1226 (100.00)	962 (100.00)	280 (100.00)	—
Troponin T (ng/mL), Median (IQR); n observed	0.0945 (0.049–0.179); 1202	—	—	—
Troponin I (ng/mL), Median (IQR); n observed	—	0.270 (0.130–0.570); 750	—	—
Conventional cTnI (pg/mL), Median (IQR); n observed	—	—	288.25 (140.08–590.40); 280	—
CK-MB (ng/mL), Median (IQR); n observed	—	—	18.00 (9.00–32.00); 280	—
BNP (pg/mL), Median (IQR); n observed	—	—	456.00 (180.50–965.25); 280	—
NT-proBNP (pg/mL), Median (IQR); n observed	—	—	1567.50 (765.00–3090.50); 280	—
**Treatment variables**				
Antibiotic use, n (%)	1175 (95.84)	790 (82.12)	260 (92.86)	<0.001
Vasopressor use, n (%)	750 (61.17)	485 (50.42)	172 (61.43)	<0.001
Invasive ventilation, n (%)	811 (66.15)	507 (52.70)	205 (73.21)	<0.001
**Comorbidities**				
Cerebrovascular disease, n (%)	173 (14.11)	84 (8.73)	94 (33.57)	<0.001
Diabetes, n (%)	336 (27.41)	269 (27.96)	88 (31.43)	0.399
Renal disease, n (%)	279 (22.76)	199 (20.69)	45 (16.07)	0.042
Chronic pulmonary disease, n (%)	252 (20.55)	180 (18.71)	40 (14.29)	0.051
Liver disease, n (%)	276 (22.51)	332 (34.51)	42 (15.00)	<0.001
**Laboratory parameters**				
White blood cells (×10^9^/L), Median (IQR)	15.20 (10.70–21.50)	15.00 (9.40–21.48)	12.71 (7.50–18.91)	<0.001
Hemoglobin (g/dL), Mean ± SD	9.75 ± 2.38	9.54 ± 2.01	9.65 ± 2.36	0.089
Platelets (×10^9^/L), Median (IQR)	150.00 (86.25–222.75)	151.00 (86.00–243.75)	159.00 (97.00–236.50)	0.209
Creatinine (mg/dL), Median (IQR)	1.80 (1.10–3.30)	1.60 (1.00–2.80)	1.36 (0.80–2.60)	<0.001
BUN (mg/dL), Median (IQR)	33.00 (20.00–54.00)	31.00 (19.00–52.00)	32.24 (21.37–62.17)	0.061
ALT (U/L), Median (IQR)	49.00 (22.00–111.76)	48.34 (26.00–104.86)	32.66 (15.00–72.25)	<0.001
AST (U/L), Median (IQR)	78.21 (36.04–200.10)	71.00 (38.03–153.00)	41.34 (22.75–95.23)	<0.001
Total bilirubin (mg/dL), Median (IQR)	0.90 (0.50–2.10)	1.90 (0.80–4.10)	0.91 (0.47–2.16)	<0.001
PT (s), Median (IQR)	15.60 (13.40–20.78)	16.90 (14.50–22.00)	16.30 (14.70–18.72)	<0.001
PTT (s), Median (IQR)	36.05 (29.80–51.58)	37.70 (30.72–51.30)	41.95 (35.98–49.02)	<0.001
INR, Median (IQR)	1.40 (1.20–1.90)	1.50 (1.30–2.10)	1.37 (1.21–1.62)	<0.001
**Vital signs**				
Heart rate (beats/min), Mean ± SD	91.18 ± 17.49	94.63 ± 17.66	96.37 ± 18.56	<0.001
Mean blood pressure (mmHg), Mean ± SD	76.45 ± 10.92	73.49 ± 9.60	79.24 ± 11.70	<0.001
Respiratory rate (breaths/min), Median (IQR)	13.00 (10.00–16.00)	13.00 (11.00–16.00)	17.00 (14.00–20.00)	<0.001
Temperature (°C), Mean ± SD	37.50 ± 1.04	37.71 ± 1.00	37.18 ± 0.85	<0.001
Lactate (mmol/L), Median (IQR)	2.80 (1.80–5.40)	2.70 (1.80–4.40)	2.60 (1.52–3.98)	0.001
pH, Mean ± SD	7.24 ± 0.15	7.28 ± 0.13	7.37 ± 0.13	<0.001
Glucose (mg/dL), Median (IQR)	182.00 (139.00–256.00)	162.00 (125.00–217.00)	162.00 (130.65–205.65)	<0.001
Urine output (mL/day), Median (IQR)	1100.00 (475.00–2075.00)	1251.00 (641.50–2155.00)	1000.00 (553.75–1642.50)	<0.001
**Outcome**				
Hospital mortality, n (%)	423 (34.50)	281 (29.21)	67 (23.93)	0.001

**Note:** Continuous variables are presented as mean ± standard deviation (SD) or median (interquartile range, IQR) according to data distribution, and categorical variables are expressed as counts (percentages).Comparisons among groups were performed using one-way analysis of variance (ANOVA) or Kruskal–Wallis test for continuous variables, and chi-square test for categorical variables.MIMIC-IV was used as the development cohort, while MIMIC-III and the local cohort served as external validation datasets. Cardiac biomarkers are reported as assay-specific first-24-h concentrations; no cross-cohort P values are shown because assays, units, and reference-limit conventions differed. CK-MB and BNP/NT-proBNP were available only in the local cohort and are descriptive rather than eligibility or cross-cohort predictor variables.

**Fig 1:**
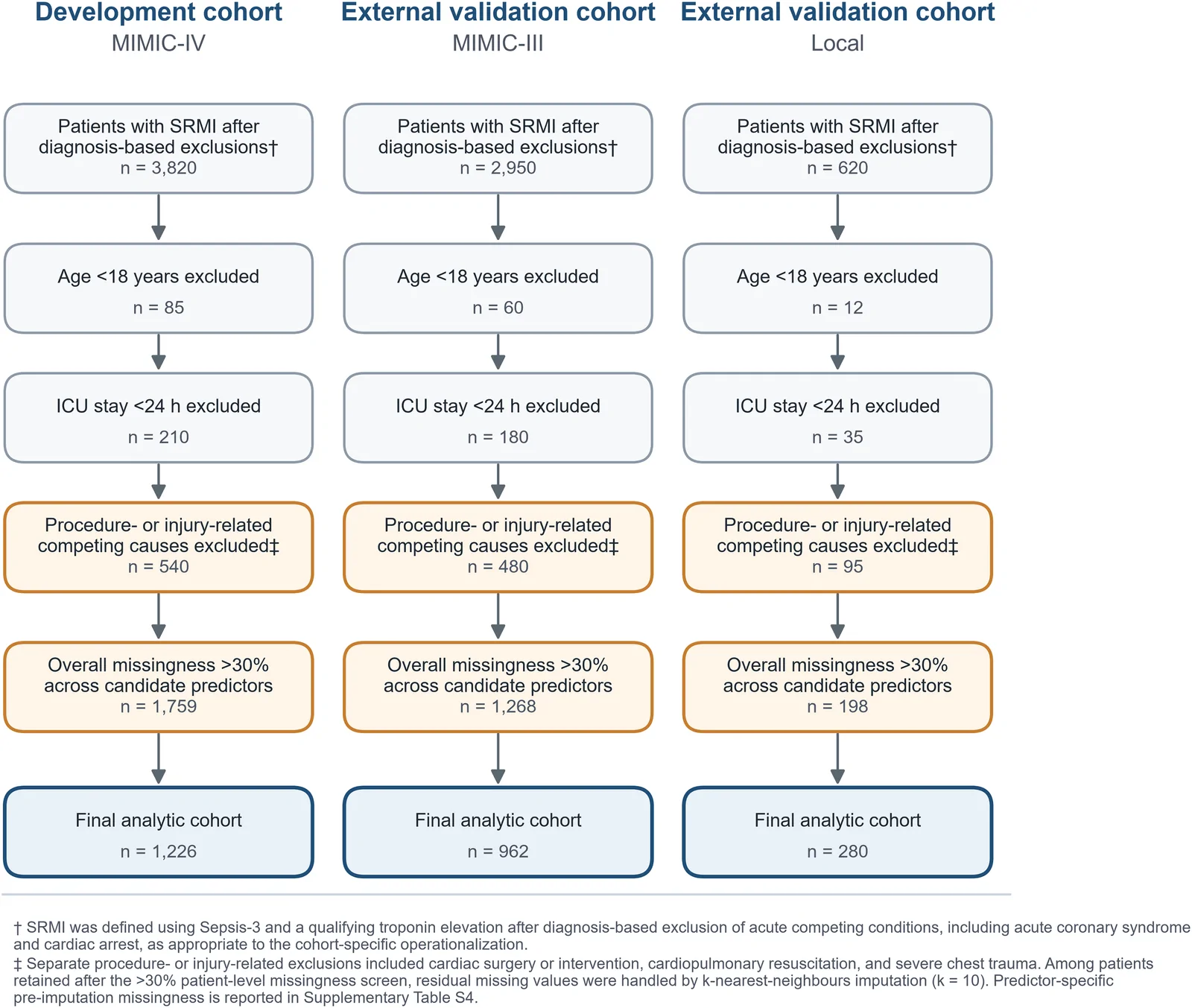
Flowchart of patient selection for the development and external validation cohorts. SRMI was defined using Sepsis-3 and a qualifying troponin elevation after diagnosis-based exclusion of acute competing conditions. Procedure- or injury-related competing causes were excluded separately. Patients with >30% overall missingness across candidate predictors were excluded; residual missing values among retained patients were imputed using k-nearest neighbours (k = 10). Legend: The starting boxes show patients with SRMI after diagnosis-based exclusion of acute competing conditions. Procedure- or injury-related competing causes, including cardiac surgery or intervention, cardiopulmonary resuscitation, and severe chest trauma, were subsequently excluded as a separate step. Patients with >30% overall missingness across candidate predictors were then excluded; no individual candidate predictor was required to be complete. Residual missing values among retained patients were imputed using k-nearest neighbours (k = 10).

With respect to treatment variables, the use of invasive mechanical ventilation was more frequent in the local cohort (73.21%) compared to MIMIC-IV (66.15%) and MIMIC-III (52.70%) (P < 0.001). Vasopressor use also varied significantly across cohorts, whereas antibiotic use was consistently high but still showed statistically significant differences.

Marked heterogeneity was also observed in comorbidities. Specifically, the prevalence of cerebrovascular disease was substantially higher in the local cohort (33.57%) than in the MIMIC cohorts, and liver disease was more common in MIMIC-III (34.51%). Renal disease showed modest differences among groups, whereas diabetes prevalence was comparable.

Laboratory parameters and vital signs showed further inter-cohort variability. Patients in MIMIC-III had higher total bilirubin levels, and the local cohort exhibited lower liver enzyme levels (ALT and AST). Coagulation parameters, including prothrombin time (PT), partial thromboplastin time (PTT), and international normalized ratio (INR), differed significantly across cohorts. Additionally, the local cohort presented with higher respiratory rates and mean blood pressure, as well as relatively higher pH values, suggesting differences in physiological status and clinical management. Hospital mortality was highest in the development cohort (34.50%), followed by MIMIC-III (29.21%) and the local cohort (23.93%) (P = 0.001). Overall, substantial differences in demographic characteristics, disease severity, treatment patterns, and laboratory profiles were observed between cohorts, indicating notable inter-cohort heterogeneity.

### 3.2 Feature selection results

As the number of included variables increased, model performance improved and reached its peak cross-validated AUC of 0.823 with 30 variables; inclusion of all 31 candidate variables yielded a comparable AUC of 0.819 (Fig 2).

**Fig 2:**
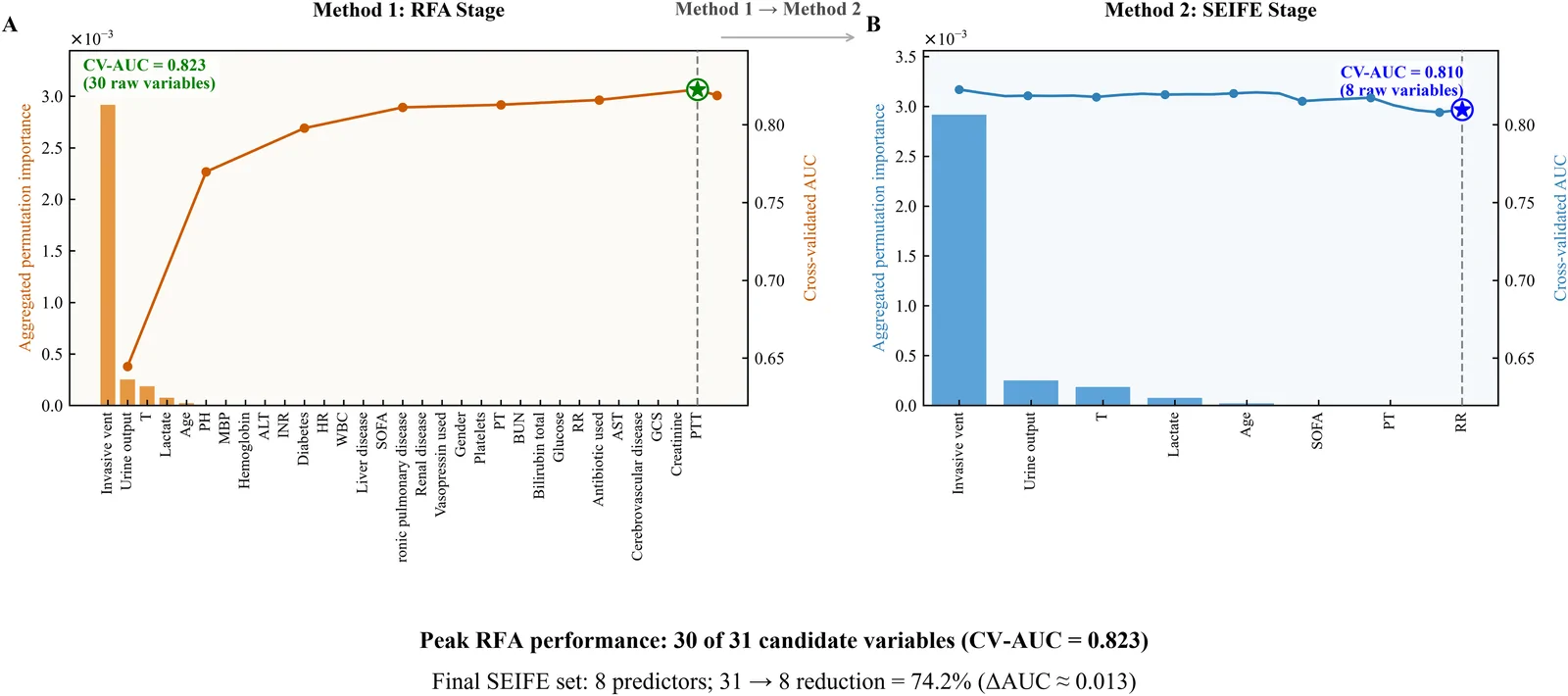
Feature selection process based on the RFA–SEIFE strategy and corresponding changes in model performance. Model performance reaches its peak with 30 raw variables (CV-AUC = 0.823); the all-31-variable model yielded a comparable CV-AUC of 0.819.

The reduction from 31 to 8 variables corresponded to a compression ratio of approximately 74.2%.

The final selected variables included invasive mechanical ventilation, urine output, temperature, lactate, age, SOFA score, PT and RR. These variables represent key domains of critical illness, including organ dysfunction, metabolic status, hemodynamic support, and physiological response. Overall, the RFA-SEIFE framework effectively reduced feature dimensionality while preserving model discrimination, thereby improving model interpretability and potential clinical applicability.

### 3.3 Model performance

Table 2 summarizes the comparative performance of the eight candidate algorithms. Rather than reiterating all tabulated estimates, the main pattern was that tree-based ensemble models showed higher overall discrimination than the linear and distance-based alternatives. In the development cohort, random forest had the highest mean AUC (0.809 ± 0.030) and PR-AUC (0.709 ± 0.034), with XGBoost and ExtraTrees showing comparable performance. This pattern is consistent with the capacity of tree-based models to accommodate nonlinear associations and interactions among routinely collected ICU variables.

**Table 2:** Performance of candidate machine learning models in the development and external validation cohorts.

Model	Dataset	AUC	PR-AUC	Brier	Accuracy	Balanced Acc	F1	Recall	Precision
Logistic	Development	0.788 ± 0.033	0.675 ± 0.038	0.189 ± 0.011	0.728 ± 0.034	0.718 ± 0.037	0.635 ± 0.045	0.687 ± 0.063	0.592 ± 0.042
	MIMIC-III	0.784	0.641	0.193	0.699	0.699	0.576	0.701	0.489
	Local	0.692	0.478	0.333	0.457	0.613	0.445	0.910	0.295
**Random Forest**	**Development**	**0.809 ± 0.030**	**0.709 ± 0.034**	**0.172 ± 0.012**	**0.741 ± 0.031**	**0.719 ± 0.035**	**0.632 ± 0.045**	**0.646 ± 0.066**	**0.622 ± 0.044**
	**MIMIC-III**	**0.819**	**0.702**	**0.162**	**0.777**	**0.756**	**0.649**	**0.708**	**0.599**
	**Local**	**0.758**	**0.525**	**0.233**	**0.582**	**0.669**	**0.489**	**0.836**	**0.346**
ExtraTrees	Development	0.800 ± 0.029	0.694 ± 0.030	0.184 ± 0.008	0.733 ± 0.032	0.723 ± 0.033	0.640 ± 0.040	0.689 ± 0.060	0.601 ± 0.044
	MIMIC-III	0.800	0.682	0.182	0.718	0.715	0.595	0.708	0.513
	Local	0.725	0.522	0.250	0.500	0.630	0.457	0.881	0.309
XGBoost	Development	0.808 ± 0.030	0.707 ± 0.039	0.176 ± 0.013	0.737 ± 0.039	0.720 ± 0.042	0.635 ± 0.050	0.665 ± 0.072	0.611 ± 0.052
	MIMIC-III	0.814	0.693	0.170	0.755	0.744	0.631	0.719	0.563
	Local	0.742	0.511	0.261	0.557	0.663	0.483	0.866	0.335
SVM	Development	0.802 ± 0.030	0.690 ± 0.039	0.169 ± 0.010	0.745 ± 0.022	0.679 ± 0.021	0.557 ± 0.034	0.464 ± 0.039	0.700 ± 0.058
	MIMIC-III	0.803	0.644	0.157	0.788	0.706	0.584	0.509	0.684
	Local	0.690	0.451	0.218	0.629	0.633	0.453	0.642	0.350
KNN	Development	0.782 ± 0.030	0.662 ± 0.032	0.177 ± 0.010	0.737 ± 0.020	0.656 ± 0.026	0.507 ± 0.050	0.395 ± 0.057	0.718 ± 0.053
	MIMIC-III	0.797	0.674	0.157	0.785	0.693	0.562	0.473	0.693
	Local	0.749	0.599	0.172	0.771	0.681	0.515	0.508	0.523
MLP	Development	0.786 ± 0.031	0.673 ± 0.037	0.174 ± 0.012	0.746 ± 0.020	0.687 ± 0.027	0.572 ± 0.046	0.496 ± 0.060	0.682 ± 0.038
	MIMIC-III	0.804	0.661	0.154	0.794	0.699	0.571	0.470	0.729
	Local	0.698	0.508	0.189	0.679	0.594	0.392	0.433	0.358
AdaBoost	Development	0.797 ± 0.033	0.690 ± 0.041	0.193 ± 0.005	0.752 ± 0.020	0.689 ± 0.024	0.574 ± 0.040	0.486 ± 0.052	0.707 ± 0.053
	MIMIC-III	0.794	0.651	0.187	0.784	0.681	0.540	0.434	0.714
	Local	0.701	0.455	0.218	0.657	0.621	0.435	0.552	0.359

**Note:** Values in the development cohort are presented as mean ± standard deviation based on repeated cross-validation. External validation results are reported for the MIMIC-III and local cohorts. AUC, area under the receiver operating characteristic curve; PR-AUC, area under the precision–recall curve; Brier, Brier score; Balanced Acc, balanced accuracy.

In external validation, random forest achieved an AUC of 0.819 (bootstrap 95% CI, 0.787-0.848), a PR-AUC of 0.702 (0.657-0.751), and a Brier score of 0.147 (0.136-0.158) in MIMIC-III. In the local cohort, the corresponding estimates were 0.758 (0.693-0.816), 0.525 (0.435-0.630), and 0.196 (0.176-0.217), respectively (S5 Table). The wider intervals in the local cohort, which included only 67 in-hospital deaths, indicate greater estimation uncertainty. Accordingly, these findings provide independent external evaluation of a fixed predictor set rather than evidence of broad generalizability; they should be interpreted alongside the observed cross-cohort distributional differences and the need for setting-specific evaluation and probability updating.

Calibration performance also varied among models. In particular, the random forest model demonstrated relatively lower Brier scores in both external cohorts (0.162 in MIMIC-III and 0.233 in the local cohort), although evidence of miscalibration was observed, particularly in the local dataset. The random forest model was selected as the primary model on the basis of its discrimination, balanced classification performance, and comparatively consistent external performance.

In the prespecified benchmark comparison, the random forest model outperformed SOFA across all cohorts (S5 Table). The paired AUC increment was 0.181 (95% CI, 0.147 to 0.216; DeLong P < 0.001) in the development cohort, 0.082 (95% CI, 0.053 to 0.109; P < 0.001) in MIMIC-III, and 0.098 (95% CI, 0.012 to 0.182; P = 0.022) in the local cohort. Relative to SOFA, random forest also yielded positive continuous NRI and IDI in development (NRI, 0.760 [95% CI, 0.658 to 0.862]; IDI, 0.213 [0.189 to 0.238]; both bootstrap P < 0.001) and MIMIC-III (NRI, 0.449 [0.318 to 0.576]; IDI, 0.132 [0.108 to 0.157]; both P < 0.001). In the local cohort, IDI remained positive (0.095 [0.034 to 0.153]; P = 0.004), whereas NRI was not statistically significant (0.227 [−0.055 to 0.494]; P = 0.119). These comparisons provide a severity-score benchmark but do not establish clinical benefit or replace prospective impact evaluation.

### 3.4 Model recalibration

Given the observed miscalibration of the random forest model in external cohorts, particularly under distributional shift, we applied the fixed development-derived logistic mapping (alpha = -0.414; beta = 1.156) estimated from repeated out-of-fold development probabilities. In the development cohort, the calibration intercept, slope, and Brier score were -0.452, 2.301, and 0.118 before application and 0.372, 1.992, and 0.109 after application, respectively (S6 Table). The post-mapping development values were not expected to equal 0 and 1 because the mapping was fitted to out-of-fold predictions but evaluated here on probabilities from the model refitted on the full development cohort. In MIMIC-III, the calibration intercept and slope were -0.635 and 1.249 before recalibration, consistent with risk overestimation and under-dispersion of predicted probabilities; after application, they shifted to -0.188 and 1.081. In the local cohort, the intercept and slope changed from -1.498 and 1.093 to -1.107 and 0.946, respectively. The lower event rates in the external cohorts (29.2% in MIMIC-III and 23.9% in the local cohort) than in the development cohort (34.5%) are consistent with this risk-overestimation pattern, because a model fitted in a higher-risk development population may overestimate risk when transported to lower-risk populations. Brier scores improved from 0.162 to 0.147 in MIMIC-III and from 0.233 to 0.196 in the local cohort. Because the transformation was monotonic, discrimination was unchanged. Percentile bootstrap 95% confidence intervals for the external calibration intercepts and slopes are reported in S6 Table.

Brier scores decreased from 0.162 to 0.147 in MIMIC-III and from 0.233 to 0.196 in the local cohort, indicating better agreement between predicted and observed risks. Calibration curves (Fig 3E) visually confirmed these findings as well, demonstrating a closer alignment between predicted and observed risks after recalibration, particularly in higher-risk regions. Importantly, recalibration primarily improved probability estimation without materially affecting discrimination performance, suggesting that the underlying model retained its ranking ability while achieving more reliable risk quantification.

**Fig 3:**
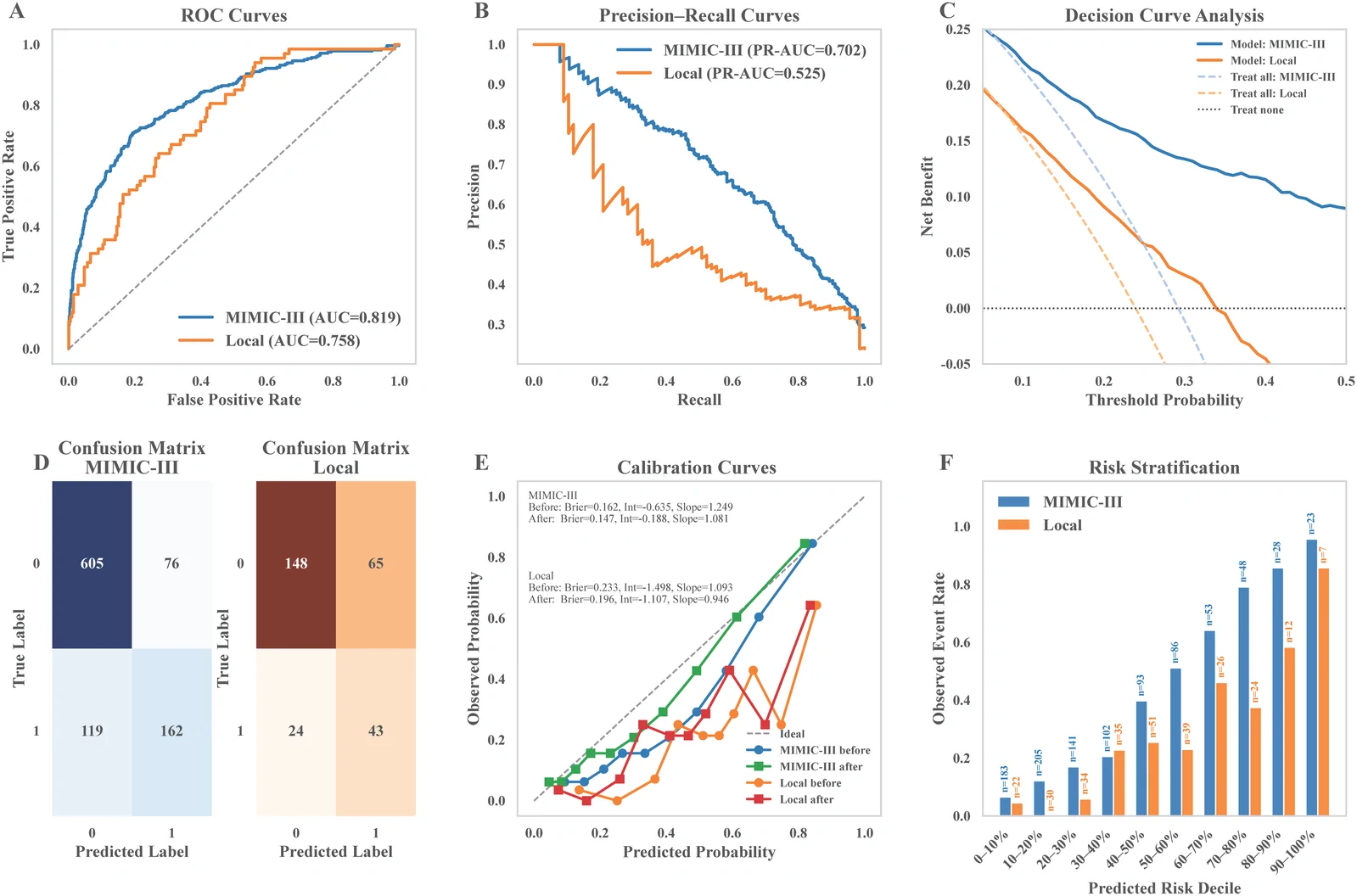
Comprehensive evaluation of the final model in external validation cohorts. Legend:(A) Receiver operating characteristic (ROC) curves showing the discriminative performance of the model in the MIMIC-III and local validation cohorts; (B) Precision–recall curves evaluating model performance under class imbalance; (C) Decision curve analysis (DCA) demonstrating the net clinical benefit across a range of threshold probabilities; (D) Confusion matrices illustrating classification performance in the external validation cohorts; (E) Calibration curves comparing predicted and observed probabilities before and after recalibration, with Brier score, calibration intercept, and slope reported; (F) Risk stratification analysis showing observed event rates across predicted risk deciles.

### 3.5 Model interpretability using SHAP

We used SHAP analysis to characterize the contribution of each feature to global and individual model predictions. In the MIMIC-III validation cohort (Fig 4A), PT, invasive ventilation, lactate, temperature, and urine output were identified as the most influential predictors, as indicated by their higher mean absolute SHAP values. A similar ranking pattern was observed in the local cohort (S1 Fig, panel A), suggesting that feature importance remained consistent across different populations.

**Fig 4:**
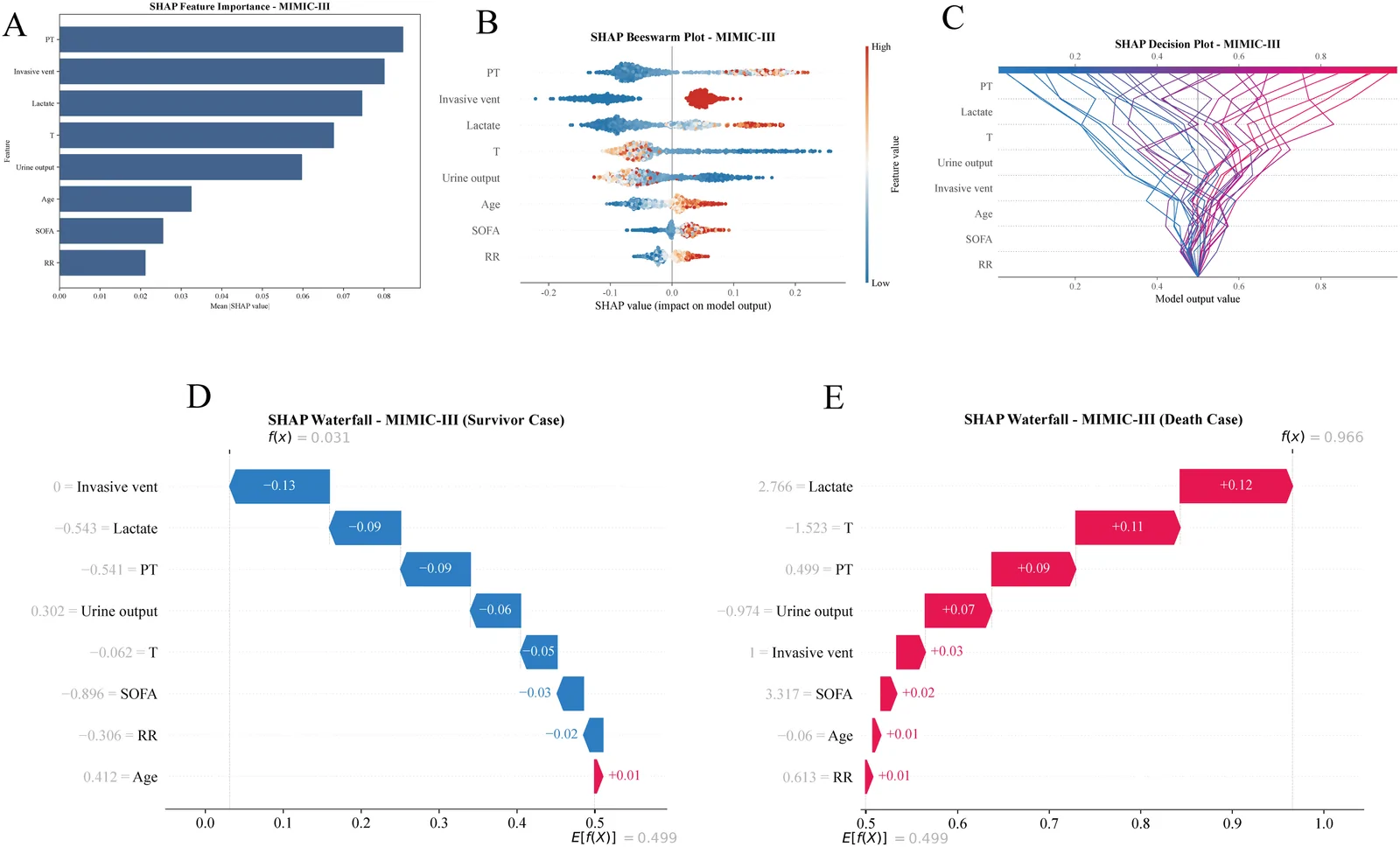
SHAP-based model interpretability analysis in the MIMIC-III validation cohort. Legend:(A) SHAP feature importance plot showing the mean absolute SHAP values of each variable, reflecting their overall contribution to model output. (B) SHAP summary (beeswarm) plot illustrating the distribution and direction of feature effects, where each point represents an individual sample and color indicates feature value. (C) SHAP decision plot demonstrating the cumulative contribution of features to individual predictions across multiple samples. (D–E) SHAP waterfall plots presenting representative individual cases, including a low-risk survivor (D) and a high-risk non-survivor (E), illustrating how feature contributions drive model predictions at the individual level.

The SHAP summary plot (Fig 4B) further illustrated the direction and magnitude of feature effects. Elevated PT and lactate levels were associated with increased mortality risk, whereas higher urine output was associated with lower predicted risk. Invasive ventilation was consistently associated with higher predicted risk, reflecting its role as a surrogate of disease severity. In addition, temperature exhibited a nonlinear relationship, with lower values contributing to increased risk. These patterns were also largely reproduced in the local cohort (S1 Fig, panel B), supporting the stability of model behavior.

Further insights into nonlinear relationships were obtained from SHAP dependence plots. In the MIMIC-III cohort (S2 Fig), lactate demonstrated a threshold-like effect, with risk increasing markedly beyond moderate levels. Urine output showed a monotonic inverse association with mortality risk, and PT exhibited a positive nonlinear relationship. Temperature displayed a U-shaped or inverse pattern, suggesting that hypothermia may be associated with worse outcomes. These nonlinear patterns were similarly observed in the local cohort (S3 Fig), with minor distributional differences likely reflecting variation in patient characteristics and clinical practice across datasets. Interaction effects, particularly involving invasive ventilation, were also identified, indicating that the impact of certain variables may depend on the overall severity of illness.

At the individual level, SHAP decision plots (Fig 4C) revealed heterogeneous prediction trajectories across patients, and representative waterfall plots further illustrated how individual feature contributions combined in these predictions. In the survivor case (Fig 4D), lower lactate, shorter PT, and absence of invasive ventilation contributed to reduced predicted risk. In contrast, in the nonsurvivor case (Fig 4E), elevated lactate (+0.120), abnormal temperature (+0.110), and prolonged PT (+0.090) were the primary drivers of increased risk. Similar patterns were observed in the local cohort (S1 Fig, panels D-E), further supporting the consistency of individual-level explanations.

Overall, SHAP analysis identified clinically interpretable model behavior, but feature-attribution patterns do not by themselves establish generalizability or clinical benefit. Their value in this study is to make the basis of individual model predictions transparent and to support comparison of explanatory patterns across external cohorts.

### 3.6 Sensitivity analyses: Distribution shift, harmonization, and class weighting

In a class-weighting sensitivity analysis using the same predictors and fixed hyperparameters, removal of class_weight = balanced did not materially change discrimination but improved raw probability calibration (S7 Table). In MIMIC-III, unweighted versus weighted random forest yielded AUC 0.820 versus 0.819 (difference, 0.002; 95% CI, -0.002 to 0.005), PR-AUC 0.705 versus 0.702 (0.003; -0.004 to 0.009), and Brier score 0.148 versus 0.162 (-0.014; -0.016 to -0.011); the calibration intercept moved from -0.635 to -0.119, whereas the slope remained similar (1.249 versus 1.256). In the local cohort, AUC was 0.755 versus 0.758 (-0.003; -0.012 to 0.006), PR-AUC 0.523 versus 0.525 (-0.002; -0.019 to 0.015), and Brier score 0.191 versus 0.233 (-0.041; -0.047 to -0.036); the intercept moved from -1.498 to -1.037 and the slope from 1.093 to 1.069. The development OOF-selected threshold was 0.295 for the unweighted model and 0.384 for the weighted model; when transported unchanged, balanced accuracy was similar in MIMIC-III (0.722 versus 0.726) and the local cohort (0.661 versus 0.656).

We used PSI and development-anchored harmonization as a sensitivity analysis to assess the model under cross-cohort distributional differences. Substantial distributional shifts were observed between the development cohort and external cohorts prior to harmonization (S4 Fig, panel B). In particular, SOFA score and respiratory rate exhibited high PSI values, indicating pronounced heterogeneity across datasets. However, after applying development-anchored harmonization, PSI values were reduced for most variables (S4 Fig, panel A), suggesting improved alignment of feature distributions.

We re-evaluated the model using harmonized data to assess the effect of distributional alignment on performance (S5 Fig). Here a slight decrease in discrimination was observed (ΔAUC range: -0.003 to -0.023), but calibration performance improved, particularly in the local cohort (ΔBrier up to -0.035). These findings indicate that although harmonization may reduce apparent discrimination, it enhances probability calibration.

Despite notable distributional shifts across cohorts, discrimination changed only modestly after the sensitivity harmonization. This finding does not establish generalizable relationships; rather, it indicates that simple distribution alignment did not materially alter discrimination in these data. The primary analysis therefore used the original data, whereas results after harmonization were retained as a sensitivity analysis.

## 4 Discussion

In this multicohort study, we evaluated a parsimonious machine-learning framework for in-hospital mortality prediction in SRMI across two independent external cohorts. The contribution is not the novelty of the individual algorithms; rather, it is the joint evaluation of discrimination, probability calibration, distributional shift, and model interpretation in settings with differing case mix. This approach allows performance differences across cohorts to be interpreted in relation to both distributional heterogeneity and probability miscalibration.

SRMI represents a complex systemic condition driven by multiple pathophysiological processes, including inflammatory activation, microcirculatory dysfunction, oxidative stress, and mitochondrial impairment, which collectively contribute to myocardial energy dysregulation and contractile dysfunction [5,10–13]. In sepsis, myocardial injury is strongly associated with adverse outcomes and increased mortality [6,7]. However, reliance on single biomarkers is not sufficient to predict mortality. Although cardiac troponins and natriuretic peptides are widely used for detecting myocardial injury, their prognostic performance for mortality prediction is limited when used in isolation [8,16,17]. These limitations support the need for integrative approaches that incorporate multiple clinical dimensions.

Machine learning provides a flexible framework capable of capturing complex nonlinear relationships and interactions between variables. Previous studies have demonstrated that machine-learning-based models can outperform traditional statistical approaches in predicting sepsis outcomes [18–21]. In particular, ensemble learning methods have shown advantages in handling heterogeneous and high-dimensional clinical data [19]. However, despite promising internal performance, many models exhibit reduced generalizability when applied to external datasets, largely due to differences in patient populations and clinical practice patterns [22].

In the present study, the random forest model showed the strongest overall discrimination among the evaluated algorithms. Tree-based ensemble methods can accommodate nonlinear associations and interactions without requiring strong parametric assumptions [29]. However, the lower performance observed in the local cohort and the detected distributional differences indicate that performance estimates should be interpreted as setting dependent rather than as evidence of universal superiority. Discrimination alone is also insufficient to establish clinical reliability; accurate risk estimation is equally important. In direct comparison with SOFA, the random forest model showed higher discrimination, precision-recall performance, and lower Brier scores in all three cohorts. This finding supports added prognostic information beyond a single routinely used severity score, while not establishing superiority over APACHE II because that score could not be harmonized across the three cohorts.

SHAP analysis clarified how the final random forest model used the eight predictors. In MIMIC-III, prothrombin time, invasive ventilation, lactate, temperature, and urine output had the largest mean absolute SHAP values. Higher prothrombin time and lactate, and invasive ventilation, generally contributed to higher predicted mortality risk, whereas higher urine output generally contributed to lower predicted risk. These patterns are clinically plausible markers of coagulopathy, organ-support intensity, hypoperfusion, and physiologic reserve. SHAP improves transparency of the prediction mechanism but does not establish causality or clinical utility.

A major strength of this study lies in its explicit evaluation of calibration and implementation of recalibration strategies. Although discrimination performance remained relatively stable across datasets, calibration was substantially degraded in external cohorts prior to recalibration. This observation is consistent with previous reports that indicate that predictive models often exhibit systematic miscalibration when applied to new populations [25]. Such discrepancies are typically driven by differences in baseline risk and data distribution. With a fixed logistic mapping derived from the development cohort, Brier scores decreased and calibration slopes moved toward 1, consistent with improved probability calibration. These findings emphasize that recalibration is a critical step for translating prediction models into clinical practice.

The class-weighting sensitivity analysis supports interpreting class weighting as one contributor to, rather than the sole explanation for, the observed probability-scale mismatch. Removing class weighting improved the raw external Brier score and moved the calibration intercept toward zero without materially changing external discrimination; however, calibration slopes remained above 1 and cross-cohort baseline-risk differences persisted. Thus, class weighting involves a discrimination-calibration trade-off that should be evaluated for the intended use setting. Threshold tuning changes classification labels for decisions but cannot by itself correct probability calibration.

The weighted random forest was retained as the primary model because it was selected by the prespecified primary model-comparison workflow, whereas the unweighted analysis was a post hoc sensitivity analysis rather than a model re-selection exercise. Moreover, after the fixed development-derived recalibration, the MIMIC-III Brier score of the weighted model was 0.147, comparable to 0.148 for the unweighted model before recalibration. Thus, the sensitivity analysis identifies a probability-calibration consideration but does not establish that an unweighted model should replace the prespecified primary model.

Dataset shift was further quantified using the PSI, which demonstrated substantial distributional differences between the development and external cohorts. This is consistent with prior evidence on the impact of temporal and population heterogeneity on model performance [24]. Although development-anchored harmonization reduced PSI values, it did not consistently improve discrimination, suggesting that simple distribution alignment may not be sufficient to address generalizability challenges alone.

From a clinical perspective, the proposed model may support early SRMI risk stratification, but it is not presented as a deployment-ready tool. The SHAP analyses provide transparent descriptions of how the model used observed predictors, whereas prospective evidence is required to establish whether model-guided decisions improve care or outcomes. Clinical utility therefore depends on prospective validation, appropriate calibration or updating in the intended setting, and assessment of implementation consequences.

Several limitations should be acknowledged. First, the retrospective design introduces the possibility of residual confounding and selection bias. Second, although multicohort validation was performed, the local cohort was derived from a single center and included 67 in-hospital deaths; its wider bootstrap intervals indicate limited precision and preclude claims of broad generalizability. Third, the restricted eight-variable predictor set and incomplete availability of potentially informative variables, such as echocardiographic parameters and longitudinal data, may have constrained discrimination. Fourth, the SRMI phenotype was operationalized using cohort-specific assays, reference-limit information, and ascertainment procedures. In MIMIC, diagnosis and procedure codes were applied as admission-level proxies and did not permit reliable temporal ordering for all competing causes; the local cohort used conventional cTnI and manual chart review. The cohorts should therefore not be interpreted as diagnostically identical, and assay-era and ascertainment differences may have contributed to the observed case-mix and distributional shifts. Fifth, the observed distributional differences mean that a model developed in one setting may require local evaluation, recalibration, or updating before use elsewhere. Sixth, exclusion of patients with >30% overall missingness across candidate predictors may have introduced selection bias that KNN imputation among retained patients cannot remove. KNN also cannot eliminate between-cohort distributional differences and may influence transported probability estimates. Accordingly, the PSI, discrimination, and calibration results should be interpreted as properties of the complete preprocessing-and-modeling workflow, rather than of the model alone. Prospective multicenter development and validation, richer longitudinal predictors, and repeated model updating should therefore be evaluated as future improvements rather than inferred from the present analysis.

## 5 Conclusion

In this multicohort study, we developed and externally evaluated a parsimonious machine-learning model for in-hospital mortality in SRMI. Using two-stage feature selection and systematic model comparison, an eight-variable random forest model showed moderate discrimination in two external cohorts. The smaller single-center local cohort provided an independent but imprecise evaluation rather than proof of broad generalizability. Recalibration improved probability estimation, but the observed cross-cohort heterogeneity indicates that absolute risk estimates may require local assessment and updating.

SHAP analysis identified prothrombin time, invasive ventilation, lactate, temperature, urine output, age, SOFA score, and respiratory rate as contributors to model predictions. The study provides an interpretable external-evaluation framework for early SRMI risk stratification using routinely available ICU data; prospective multicenter validation is needed before clinical deployment.

## Supporting information

S1 Fig SHAP-based model interpretability analysis in the local validation cohort. Legend: (A) SHAP feature importance plot showing the mean absolute SHAP values of each variable, reflecting their overall contribution to model output in the local cohort. (B) SHAP summary (beeswarm) plot illustrating the distribution and direction of feature effects, where each point represents an individual sample and color indicates feature value. (C) SHAP decision plot demonstrating the cumulative contribution of features to individual predictions across multiple samples. (D–E) SHAP waterfall plots presenting representative individual cases, including a low-risk survivor (D) and a high-risk non-survivor (E), illustrating how feature contributions drive model predictions at the individual level.
(TIF)

S2 Fig SHAP dependence plots for key variables in the MIMIC-III cohort. Legend: SHAP dependence plots illustrating the relationships between key variables and their corresponding SHAP values in the MIMIC-III cohorts. Each panel represents one variable, showing the effect of the feature value on model output. Color indicates the value of an interacting feature, highlighting potential interaction effects. These plots provide detailed insights into nonlinear relationships and interaction patterns between predictors and model predictions.
(TIF)

S3 Fig SHAP dependence plots for key variables in the local cohort. Legend: SHAP dependence plots illustrating the relationships between key variables and their corresponding SHAP values in the local cohorts. Each panel represents one variable, showing the effect of the feature value on model output. Color indicates the value of an interacting feature, highlighting potential interaction effects. These plots provide detailed insights into nonlinear relationships and interaction patterns between predictors and model predictions.
(TIF)

S4 Fig Population stability index before and after development-anchored harmonization. Legend: (A) Population stability index after development-anchored harmonization. (B) Population stability index before development-anchored harmonization. PSI values quantify feature-distribution differences between the development cohort and the external cohorts (MIMIC-III and local). Continuous variables used development-cohort decile bins, whereas categorical variables used category frequencies. Values <0.10, 0.10-0.25, and >0.25 were interpreted descriptively as stable, moderate, and substantial shift, respectively. Reduced PSI after harmonization indicates altered distributional alignment; it does not by itself demonstrate improved transportability. PSI results were evaluated as distribution-shift diagnostics. Reduced PSI reflects alignment to development-derived bins and was not interpreted as proof of improved transportability.
(TIF)

S5 Fig Changes in model performance after development-anchored harmonization. Legend: Changes in model performance after development-anchored harmonization in the MIMIC-III and local cohorts. The left panel shows the change in AUC (ΔAUC), and the right panel shows the change in Brier score (ΔBrier). Negative ΔBrier values indicate improved calibration performance. Overall, harmonization led to slight reductions in discrimination but improved calibration, particularly in the local cohort.
(TIF)

S1 Table Database-specific operationalization of the SRMI definition. (DOCX)

S2 Table Variable-level definitions and first-24-hour aggregation rules. (DOCX)

S3 Table Hyperparameter search spaces and selected configurations for the eight-model comparison. (DOCX)

S4 Table Missing data percentages for candidate predictors in the development and external validation cohorts. (DOCX)

S5 Table Direct benchmark and incremental-value comparison of random forest versus SOFA score. (DOCX)

S6 Table Logistic recalibration coefficients and calibration performance before and after fixed mapping. (DOCX)

S7 Table Sensitivity analysis of random-forest class weighting. (DOCX)

S1 Checklist TRIPOD AI checklist. (DOCX)
