# Supplementary material for: A machine learning model for in-hospital mortality prediction in patients with sepsis-related myocardial injury
Source: PLOS Digit Health. 2026 Oct 12;5(10):e0001758. doi: 10.1371/journal.pdig.0001758 (PMC13657301; doi:10.1371/journal.pdig.0001758)
Supplement: Supplementary file 1 [file pdig.0001758.s006.docx]

**S1 Table. Database-specific operationalization of the SRMI definition**

| **Cohort** | **Cardiac-marker source/assay** | **Eligibility rule** | **Time window** | **Exclusion method** |
| --- | --- | --- | --- | --- |
| MIMIC-IV | labevents: Troponin T (51003), Troponin I (51002), and hs-Troponin I (52642) | Value > row-level ref_range_upper when available and >0; otherwise laboratory abnormal flag. No common fixed cutoff. | First ICU 24 h and within ±24 h of operational sepsis time | Admission-level ICD-9/10 diagnosis and procedure codes; no chart review. ACS/AMI, PE, myocarditis, cardiac arrest, cardiac surgery/intervention, CPR, and severe chest trauma excluded. |
| MIMIC-III | labevents: Troponin I (51002) and Troponin T (51003) | Laboratory abnormal flag. Stable row-level URL unavailable; no common fixed cutoff. | First ICU 24 h and within ±24 h of operational sepsis time | Admission-level ICD-9 diagnosis and procedure codes; no chart review. Same competing-cause categories excluded. |
| Local | Beckman Coulter Access AccuTnI+3 conventional cTnI; CK-MB and BNP/NT-proBNP first-24-h maxima retained for characterization | At least one conventional cTnI >40 pg/mL. CK-MB and BNP/NT-proBNP did not determine eligibility. | First ICU 24 h | Manual medical-record review excluded ACS and competing causes, including myocarditis, post-cardiac surgery/intervention injury, post-CPR injury, and severe chest trauma. |

*Note.* The MIMIC algorithms use a hospitalization-level proxy for code-based exclusions; timestamps sufficient to establish the temporal ordering of all procedures relative to troponin elevation were not available. MIMIC-IV ICD-9 codes: ACS/AMI 410*, 4111*, 4118*; PE 4151*; myocarditis 422*; cardiac arrest 4275*; severe chest trauma 8072*-8075*, 860*-862*, 901*; cardiac procedure 0066, 35*, 360*, 361*, 3731-3736, 3751; CPR 9960, 9963. MIMIC-IV ICD-10 codes: ACS/AMI I21*, I22*, I200, I24*; PE I26*; myocarditis I40*, I41*; cardiac arrest I46*; severe chest trauma S222*, S223*, S25*, S26*, S27*; and cardiac surgery/intervention ICD-10-PCS 02*. The MIMIC-III code sets are ICD-9 only.
