# Supplementary material for: A machine learning model for in-hospital mortality prediction in patients with sepsis-related myocardial injury
Source: PLOS Digit Health. 2026 Oct 12;5(10):e0001758. doi: 10.1371/journal.pdig.0001758 (PMC13657301; doi:10.1371/journal.pdig.0001758)
Supplement: Supplementary file 2 [file pdig.0001758.s007.docx]

**S2 Table. Variable-level definitions and first-24-hour aggregation rules**

| **Variable** | **Unit** | **First-24-h summary rule** | **Source/operational rule** |
| --- | --- | --- | --- |
| Gender | Female/male | Recorded sex | Recorded demographic sex at admission. |
| Age | years | Admission age | Age at hospital admission. |
| SOFA | points | First-24-h derived score | Derived SOFA score using data available from ICU admission to 24 h. |
| GCS | points | First-24-h derived score | Derived neurologic score using first-24-h observations. |
| Antibiotic used | Yes/no | Any first-24-h exposure | Any antibiotic exposure from ICU admission to 24 h. |
| Vasopressin used | Yes/no | Any first-24-h exposure | Any vasopressor exposure from ICU admission to 24 h. |
| Invasive vent | Yes/no | Any first-24-h exposure | Any invasive mechanical ventilation from ICU admission to 24 h. |
| Cerebrovascular disease | Yes/no | Pre-existing diagnosis | Diagnosis documented before or at admission. |
| Diabetes | Yes/no | Pre-existing diagnosis | Diagnosis documented before or at admission. |
| Renal disease | Yes/no | Pre-existing diagnosis | Diagnosis documented before or at admission. |
| Chronic pulmonary disease | Yes/no | Pre-existing diagnosis | Diagnosis documented before or at admission. |
| Liver disease | Yes/no | Pre-existing diagnosis | Diagnosis documented before or at admission. |
| WBC | 10^9/L | Maximum recorded value | Maximum from ICU admission to 24 h. |
| Hemoglobin | g/dL | Minimum recorded value | Minimum from ICU admission to 24 h. |
| Platelets | 10^9/L | Minimum recorded value | Minimum from ICU admission to 24 h. |
| Creatinine | mg/dL | Maximum recorded value | Maximum from ICU admission to 24 h. |
| BUN | mg/dL | Maximum recorded value | Maximum from ICU admission to 24 h. |
| ALT | U/L | Maximum recorded value | Maximum from ICU admission to 24 h. |
| AST | U/L | Maximum recorded value | Maximum from ICU admission to 24 h. |
| Bilirubin total | mg/dL | Maximum recorded value | Maximum from ICU admission to 24 h. |
| PT | s | Maximum recorded value | Maximum from ICU admission to 24 h. |
| PTT | s | Maximum recorded value | Maximum from ICU admission to 24 h. |
| INR | ratio | Maximum recorded value | Maximum from ICU admission to 24 h. |
| HR | beats/min | Mean recorded value | Mean of all eligible observations from ICU admission to 24 h. |
| MBP | mmHg | Mean recorded value | Mean of all eligible observations from ICU admission to 24 h. |
| RR | breaths/min | Mean recorded value | Mean of all eligible observations from ICU admission to 24 h. |
| T | degrees C | Mean recorded value | Mean of all eligible observations from ICU admission to 24 h. |
| Lactate | mmol/L | Maximum recorded value | Maximum from ICU admission to 24 h. |
| PH | ratio | Minimum recorded value | Minimum from ICU admission to 24 h. |
| Glucose | mg/dL | Maximum recorded value | Maximum from ICU admission to 24 h. |
| Urine output | mL | Cumulative first-24-h output | Total urine output from ICU admission to 24 h. |

*Note:* All time-varying predictors were restricted to ICU admission through 24 h. No information recorded after 24 h was used as a predictor. Treatment variables were included for prediction and should not be interpreted causally.
