# Supplementary material for: A machine learning model for in-hospital mortality prediction in patients with sepsis-related myocardial injury
Source: PLOS Digit Health. 2026 Oct 12;5(10):e0001758. doi: 10.1371/journal.pdig.0001758 (PMC13657301; doi:10.1371/journal.pdig.0001758)
Supplement: Supplementary file 3 [file pdig.0001758.s008.docx]

**S3 Table. Hyperparameter search spaces and selected configurations for the eight-model comparison**

| **Model** | **Hyperparameter search space** | **Class-imbalance handling** | **Selected configuration** |
| --- | --- | --- | --- |
| Logistic regression | C: 10^-3 to 10^2 (20 log-spaced values); penalty: l1, l2 | class_weight = balanced | C = 0.1274; penalty = l2 |
| Random forest | n_estimators: 200, 300, 500, 700; max_depth: 3, 5, 7, 10, None; min_samples_split: 2, 5, 10, 20; min_samples_leaf: 1, 2, 4, 8; max_features: sqrt, log2, None | class_weight = balanced | 300 trees; max_depth = None; min_samples_split = 20; min_samples_leaf = 8; max_features = log2 |
| Extra trees | n_estimators: 200, 300, 500; max_depth: None, 5, 10; min_samples_split: 2, 10, 20; min_samples_leaf: 1, 4, 8; max_features: sqrt, log2, None | class_weight = balanced | 200 trees; max_depth = None; min_samples_split = 2; min_samples_leaf = 4; max_features = sqrt |
| XGBoost | n_estimators: 150, 250, 350, 500; max_depth: 2, 3, 4, 5; learning_rate: 0.01, 0.03, 0.05, 0.1; subsample: 0.7, 0.8, 0.9, 1.0; colsample_bytree: 0.6, 0.8, 1.0; min_child_weight: 1, 3, 5, 7; reg_lambda: 0.5, 1, 2, 5 | scale_pos_weight = n_negative/n_positive | 150 trees; depth = 3; learning_rate = 0.03; subsample = 0.9; colsample = 0.8; min_child_weight = 7; reg_lambda = 2 |
| Support vector machine | C: 10^-2 to 10^2 (15 log-spaced values); gamma: scale, auto, 0.001, 0.01, 0.1, 1.0 | class_weight = balanced | C = 51.7947; gamma = 0.01 |
| K-nearest neighbours | n_neighbors: 3, 5, 7, 9, 11, 15, 21; weights: uniform, distance; p: 1, 2 | None | n_neighbors = 21; weights = distance; p = 1 |
| Multilayer perceptron | hidden_layer_sizes: (32), (64), (64,32), (128,64); alpha: 10^-5, 10^-4, 5x10^-4, 10^-3, 5x10^-3; learning_rate_init: 10^-4, 5x10^-4, 10^-3, 5x10^-3 | None | hidden_layer_sizes = (64); alpha = 0.001; learning_rate_init = 0.005 |
| AdaBoost | n_estimators: 50, 100, 200; learning_rate: 0.01, 0.1, 0.5, 1.0 | None | n_estimators = 200; learning_rate = 0.5 |

*Note.* Randomized search used stratified 5-fold cross-validation, ROC-AUC scoring, random_state = 42, and 20 sampled parameter sets for each algorithm.
