# Supplementary material for: A machine learning model for in-hospital mortality prediction in patients with sepsis-related myocardial injury
Source: PLOS Digit Health. 2026 Oct 12;5(10):e0001758. doi: 10.1371/journal.pdig.0001758 (PMC13657301; doi:10.1371/journal.pdig.0001758)
Supplement: Supplementary file 4 [file pdig.0001758.s009.docx]

**S4 Table. Missing data percentages for candidate predictors in the development and external validation cohorts**

| **Candidate predictor** | **MIMIC-IV development, %** | **MIMIC-III external, %** | **Local external, %** |
| --- | --- | --- | --- |
| Gender | 0.00 | 0.00 | 0.00 |
| Age | 0.00 | 0.00 | 0.00 |
| SOFA | 0.00 | 0.00 | 0.00 |
| GCS | 0.17 | 0.18 | 0.00 |
| Antibiotic used | 0.00 | 0.00 | 0.00 |
| Vasopressin used | 0.00 | 0.00 | 0.00 |
| Invasive vent | 0.00 | 0.00 | 0.00 |
| Cerebrovascular disease | 0.00 | 0.00 | 0.00 |
| Diabetes | 0.00 | 0.00 | 0.00 |
| Renal disease | 0.00 | 0.00 | 0.00 |
| Chronic pulmonary disease | 0.00 | 0.00 | 0.00 |
| Liver disease | 0.00 | 0.00 | 0.00 |
| WBC | 0.17 | 0.18 | 0.00 |
| Hemoglobin | 0.17 | 0.09 | 0.00 |
| Platelets | 0.17 | 0.18 | 0.00 |
| Creatinine | 0.11 | 0.18 | 0.00 |
| BUN | 0.22 | 0.27 | 0.00 |
| ALT | 19.40 | 19.40 | 0.00 |
| AST | 19.12 | 19.12 | 0.00 |
| Bilirubin total | 19.73 | 19.73 | 0.00 |
| PT | 5.25 | 2.33 | 0.00 |
| PTT | 5.65 | 2.86 | 0.00 |
| INR | 5.37 | 2.33 | 0.00 |
| HR | 0.17 | 0.18 | 0.00 |
| MBP | 0.17 | 0.18 | 0.00 |
| RR | 0.17 | 0.18 | 0.00 |
| T | 3.63 | 0.72 | 0.00 |
| Lactate | 21.86 | 17.19 | 0.00 |
| PH | 14.20 | 23.55 | 0.00 |
| Glucose | 0.11 | 0.18 | 0.00 |
| Urine output | 3.30 | 3.58 | 0.00 |
