# Supplementary material for: A machine learning model for in-hospital mortality prediction in patients with sepsis-related myocardial injury
Source: PLOS Digit Health. 2026 Oct 12;5(10):e0001758. doi: 10.1371/journal.pdig.0001758 (PMC13657301; doi:10.1371/journal.pdig.0001758)
Supplement: Supplementary file 5 [file pdig.0001758.s010.docx]

**S5 Table. Direct benchmark and incremental-value comparison of random forest versus SOFA score**

**A. Performance estimates with stratified bootstrap 95% confidence intervals**

| **Cohort** | **Approach** | **AUC (95% CI)** | **PR-AUC (95% CI)** | **Brier score (95% CI)** |
| --- | --- | --- | --- | --- |
| Development | Random forest | 0.810 (0.784-0.834) | 0.707 (0.667-0.746) | 0.171 (0.161-0.181) |
| Development | SOFA | 0.628 (0.595-0.661) | 0.464 (0.428-0.506) | 0.216 (0.210-0.222) |
| MIMIC-III | Random forest | 0.819 (0.787-0.848) | 0.702 (0.657-0.751) | 0.147 (0.136-0.158) |
| MIMIC-III | SOFA | 0.737 (0.701-0.773) | 0.562 (0.512-0.618) | 0.211 (0.200-0.220) |
| Local | Random forest | 0.758 (0.693-0.816) | 0.525 (0.435-0.630) | 0.196 (0.176-0.217) |
| Local | SOFA | 0.660 (0.579-0.738) | 0.426 (0.339-0.536) | 0.261 (0.239-0.284) |

**B. Paired discrimination and incremental-value statistics (random forest relative to SOFA)**

| **Cohort** | **ΔAUC (95% CI)** | **DeLong P** | **NRI (95% CI)** | **Bootstrap P** | **IDI (95% CI)** | **Bootstrap P** |
| --- | --- | --- | --- | --- | --- | --- |
| Development | 0.181 (0.147-0.216) | <0.001 | 0.760 (0.658-0.862) | <0.001 | 0.213 (0.189-0.238) | <0.001 |
| MIMIC-III | 0.082 (0.053-0.109) | <0.001 | 0.449 (0.318-0.576) | <0.001 | 0.132 (0.108-0.157) | <0.001 |
| Local | 0.098 (0.012-0.182) | 0.022 | 0.227 (-0.055-0.494) | 0.119 | 0.095 (0.034-0.153) | 0.004 |

*Note.* Development random-forest estimates used stored out-of-fold probabilities. SOFA used five-fold stratified out-of-fold prediction from a development-fitted one-variable logistic calibration model; this mapping was transported unchanged to the external cohorts. Percentile 95% confidence intervals were estimated using 2,000 stratified nonparametric bootstrap replicates (seed 20260803). NRI is continuous (category-free) net reclassification improvement; IDI is integrated discrimination improvement. Positive ΔAUC, NRI, and IDI favor random forest.
