# Supplementary material for: A machine learning model for in-hospital mortality prediction in patients with sepsis-related myocardial injury
Source: PLOS Digit Health. 2026 Oct 12;5(10):e0001758. doi: 10.1371/journal.pdig.0001758 (PMC13657301; doi:10.1371/journal.pdig.0001758)
Supplement: Supplementary file 6 [file pdig.0001758.s011.docx]

**S6 Table. Logistic recalibration coefficients and calibration performance before and after fixed mapping**

*OOF-fitted mapping: logit(Padj) = -0.414 + 1.156 x logit(Ppred), estimated from repeated stratified 5-fold development-cohort out-of-fold probabilities (three repetitions; n = 1,226). The fixed mapping was applied unchanged to final development-model and external-cohort probabilities.*

| **Cohort** | **n** | **Event rate, %** | **Brier before** | **Brier after** | **Intercept before** | **Intercept after** | **Slope before** | **Slope after** |
| --- | --- | --- | --- | --- | --- | --- | --- | --- |
| Development | 1,226 | 34.5 | 0.118 | 0.109 | -0.452 | 0.372 | 2.301 | 1.992 |
| MIMIC-III | 962 | 29.2 | 0.162 | 0.147 | -0.635 | -0.188 | 1.249 | 1.081 |
| Local | 280 | 23.9 | 0.233 | 0.196 | -1.498 | -1.107 | 1.093 | 0.946 |

**B. External-cohort calibration metrics with bootstrap 95% confidence intervals**

| **Cohort** | **Stage** | **Calibration intercept (95% CI)** | **Calibration slope (95% CI)** |
| --- | --- | --- | --- |
| MIMIC-III | Before | -0.635 (-0.727 to -0.550) | 1.249 (1.070 to 1.447) |
| MIMIC-III | After | -0.188 (-0.296 to -0.070) | 1.081 (0.925 to 1.261) |
| Local | Before | -1.498 (-1.748 to -1.323) | 1.093 (0.780 to 1.483) |
| Local | After | -1.107 (-1.264 to -0.992) | 0.946 (0.677 to 1.292) |

*Note.* Percentile 95% confidence intervals were estimated using 2,000 stratified nonparametric bootstrap replicates (seed 20260803).
