# Supplementary material for: A machine learning model for in-hospital mortality prediction in patients with sepsis-related myocardial injury
Source: PLOS Digit Health. 2026 Oct 12;5(10):e0001758. doi: 10.1371/journal.pdig.0001758 (PMC13657301; doi:10.1371/journal.pdig.0001758)
Supplement: Supplementary file 7 [file pdig.0001758.s012.docx]

**S7 Table. Sensitivity analysis of random-forest class weighting**

**A. Threshold-independent probability performance before recalibration**

| **Model** | **Cohort** | **n** | **Event rate, %** | **AUC** | **PR-AUC** | **Brier** | **Intercept** | **Slope** |
| --- | --- | --- | --- | --- | --- | --- | --- | --- |
| Weighted RF | Development | 1,226 | 34.5 | 0.934 | 0.883 | 0.118 | -0.452 | 2.301 |
| Weighted RF | MIMIC-III | 962 | 29.2 | 0.819 | 0.702 | 0.162 | -0.635 | 1.249 |
| Weighted RF | Local | 280 | 23.9 | 0.758 | 0.525 | 0.233 | -1.498 | 1.093 |
| Unweighted RF | Development | 1,226 | 34.5 | 0.931 | 0.879 | 0.115 | 0.553 | 2.244 |
| Unweighted RF | MIMIC-III | 962 | 29.2 | 0.820 | 0.705 | 0.148 | -0.119 | 1.256 |
| Unweighted RF | Local | 280 | 23.9 | 0.755 | 0.523 | 0.191 | -1.037 | 1.069 |

**B. External unweighted-minus-weighted performance differences**

| **Cohort** | **Metric** | **Difference (95% CI)** |
| --- | --- | --- |
| MIMIC-III | AUC | +0.002 (-0.002 to +0.005) |
| MIMIC-III | PR-AUC | +0.003 (-0.004 to +0.009) |
| MIMIC-III | Brier | -0.014 (-0.016 to -0.011) |
| Local | AUC | -0.003 (-0.012 to +0.006) |
| Local | PR-AUC | -0.002 (-0.019 to +0.015) |
| Local | Brier | -0.041 (-0.047 to -0.036) |

**C. OOF-selected threshold and external classification performance**

| **Model** | **Cohort** | **OOF threshold** | **Balanced accuracy** | **Sensitivity** | **Specificity** | **Precision** | **F1** |
| --- | --- | --- | --- | --- | --- | --- | --- |
| Weighted RF | MIMIC-III | 0.384 | 0.726 | 0.808 | 0.645 | 0.484 | 0.605 |
| Weighted RF | Local | 0.384 | 0.656 | 0.970 | 0.343 | 0.317 | 0.478 |
| Unweighted RF | MIMIC-III | 0.295 | 0.722 | 0.797 | 0.648 | 0.483 | 0.601 |
| Unweighted RF | Local | 0.295 | 0.661 | 0.955 | 0.366 | 0.322 | 0.481 |

*Note.* Weighted RF used class_weight = balanced; unweighted RF used no class weighting. Both models used the published eight predictors and fixed random-forest hyperparameters (300 trees; max_depth = None; min_samples_split = 20; min_samples_leaf = 8; max_features = log2), with identical development-derived preprocessing. Probability metrics were calculated before logistic recalibration and before threshold selection. Differences are unweighted RF minus weighted RF; negative Brier differences favor the unweighted model. Percentile 95% confidence intervals use 2,000 stratified nonparametric bootstrap replicates (seed 20260804). OOF thresholds were selected by the Youden index from repeated stratified 5-fold development-cohort probabilities (three repetitions) and were transported unchanged to both external cohorts.
