# Supplementary material for: A machine learning model for in-hospital mortality prediction in patients with sepsis-related myocardial injury
Source: PLOS Digit Health. 2026 Oct 12;5(10):e0001758. doi: 10.1371/journal.pdig.0001758 (PMC13657301; doi:10.1371/journal.pdig.0001758)
Supplement: Supplementary file 9 [file pdig.0001758.s015.docx]

**Response to Comments**

**Manuscript ID:** PDIG-D-26-00759

**Title:** A machine learning model for in-hospital mortality prediction in patients with sepsis-related myocardial injury

Dear Editor,

We thank the Editor and reviewers for their careful reading and constructive recommendations. Their comments helped us sharpen the clinical framing, make the analytical workflow more transparent, and temper the interpretation of the external-validation findings.

The revision clarifies cohort construction and first-24-hour predictor extraction; provides fuller reporting of model development, feature selection, recalibration, missing-data handling, and cross-cohort evaluation; and adds the SOFA benchmark, bootstrap uncertainty estimates, and class-weighting sensitivity analysis. We also revised the Abstract, Discussion, figures, and supplementary material to make the scope and limitations of the study clearer.

Across the revised manuscript, the model is presented as a parsimonious tool for early risk stratification rather than a replacement for established severity scores or a deployment-ready clinical tool. We have emphasized the imprecision of the single-centre local validation, the importance of calibration under distributional shift, and the need for prospective multicentre evaluation.

We have also addressed the editorial requirements relating to the Author Summary, Financial Disclosure, Ethics Statement, data and code availability, figure files, and submission consistency. Point-by-point responses are provided below.

**Editorial Requirement 1. Financial Disclosure**

Please amend the detailed Financial Disclosure statement in full sentences, including all sources of financial support, grant numbers, recipient initials, the role of funders, and any salary support from funders.

**Response:**

We have revised the Financial Disclosure statement in the manuscript as follows: "This work was supported by the Postgraduate Research & Practice Innovation Program of Jiangsu Province (SJX25-1001 to XYZ); the Natural Science Foundation of Nanjing University of Chinese Medicine (XZR2023002 to DCW); the Science and Technology Program of the Bureau of Traditional Chinese Medicine of Jiangsu Province (MS2021008 to YZ); and the Department-level Project of Jiangsu Provincial Hospital of Traditional Chinese Medicine (K25092 to DCW). The funders had no role in study design, data collection and analysis, decision to publish, or preparation of the manuscript. No authors received salary from any of the funders." This exact wording will be entered in the Financial Disclosure field of the online submission system at resubmission.

**Location: Funding, Page 28, Lines 776–784.**

**Editorial Requirement 2. Data Availability**

Please resolve the PLOS data-sharing requirement by making data underlying the findings publicly available where possible, or by providing a clear ethical or legal justification for any restriction.

**Response:**

We have revised the Data Availability Statement and publicly deposited a versioned reproducibility framework at https://github.com/zxinyuan20-hue/srmi-reproducibility-framework, archived as version 1.0.0 at Zenodo (https://doi.org/10.5281/zenodo.21786891). The public package contains the analysis framework, variable dictionary, generic cohort-query template, and synthetic demonstration workflow, but no patient-level data. MIMIC-III and MIMIC-IV remain available through PhysioNet to credentialed users under the applicable data-use agreements. The local cohort remains privacy-restricted and may be available from the corresponding authors on reasonable request and with appropriate ethical approval.

**Location: Declarations, Availability of data and materials, Page 30, Lines 829–839.**

**Editorial Requirement 3. Ethics Statement**

Please ensure that the Ethics Statement is available in its entirety at the beginning of the Methods section under the subheading "Ethics Statement."

**Response:**

We have added the complete ethics information to the beginning of the Methods section under the required "Ethics Statement" subheading, while retaining the exact approvals, data-use agreement information, waiver-of-consent information, and local ethics approval number.

**Location: Methods, Ethics Statement, Page 5, Lines 121–134.**

**Editorial Requirement 4. Separate Figure Files**

Please provide separate figure files in TIFF or EPS format and follow the PLOS figure-file requirements.

**Response:**

We have prepared separate publication-quality TIFF figure files (600 dpi, RGB) in accordance with the PLOS figure-file requirements, and we will run the PLOS NAAS workflow to confirm the final files meet the journal's technical requirements before resubmission.

**Editorial Requirement 5. Title Consistency**

Please ensure that the title in the manuscript file and the title entered in the online submission form are identical.

**Response:**

We have verified that the manuscript title and the title entered in the online submission form are identical.

**Editorial Requirement 6. Author Summary**

Please provide an Author Summary of 150-200 words between the Abstract and the Introduction that is accessible to both scientific and non-scientific readers.

**Response:**

We have added an Author Summary of 150-200 words between the Abstract and the Introduction. It explains the clinical problem, study design, main findings, and limitations in accessible language without overstating clinical readiness.

**Location: Author Summary, Page 3, Lines 54–69.**

**Comments from Reviewer #1:**

We appreciate the reviewer’s thorough assessment and constructive suggestions. The original presentation did not sufficiently communicate the study rationale, methodological framework, or contribution; these issues have been addressed throughout the revised manuscript.

**Comment 1. Abstract**

*The abstract does not adequately support the title of the manuscript. It is too long and does not describe the methodology or present the results effectively. Only the random forest model is mentioned, and the contribution of the SHAP analysis is unclear. The abstract should be improved substantially and should clearly state what this research seeks to improve and why.*

**Response:**

We appreciate this important comment. We agree that the original abstract did not sufficiently present the study rationale, complete modelling framework, or interpretation of the findings. We have therefore substantially rewritten and shortened it to provide a clearer, more balanced, and self-contained summary.

The revised abstract now states the clinical objective explicitly: to develop and externally validate a parsimonious model for early in-hospital mortality risk stratification in patients with sepsis-related myocardial injury (SRMI), while evaluating transportability across heterogeneous cohorts. It describes the three-cohort design, comparison of eight machine-learning algorithms, and RFA-SEIFE feature selection that reduced 31 candidate variables to eight routinely available predictors.

The Results now report the principal external-validation findings directly: the selected random forest model achieved an AUC of 0.819 and PR-AUC of 0.702 in MIMIC-III, and an AUC of 0.758 and PR-AUC of 0.525 in the local cohort. We also clarify that SHAP quantifies the direction and magnitude of individual predictor contributions, whereas PSI characterizes cross-cohort distributional shift. These analyses complement the primary performance evaluation.

Finally, we revised the conclusion to avoid implying universal superiority over existing approaches. The contribution of this work is the combined assessment of discrimination, calibration, clinical utility, interpretability, and distributional shift in two independent external cohorts. We believe that these revisions make the abstract consistent with the title and the full scope of the manuscript.

**Location: Abstract, Page 1, Line 19 to Page 2, Line 46.**

**Comment 2. Introduction**

*The Introduction is well written.*

**Response:**

We appreciate this positive assessment and the recognition that the Introduction provides an appropriate clinical context and rationale for the study. No substantive revision was required in response to this comment; the final paragraph was nevertheless reviewed during the final consistency check to confirm that the study aim and contribution remain aligned with the revised Abstract, Methods, Results, and Discussion.

**Comment 3. Methods**

*The Methods section is too long and contains unnecessary detail, whereas the machine-learning methodology is introduced too late and is insufficiently described. The eight basic machine-learning methods are well established in medical applications.*

**Response:**

We appreciate this important observation. We agree that the current Methods section does not provide an optimal reading sequence: some generic preprocessing details are overly prominent, whereas the machine-learning workflow, which is central to this study, should be introduced earlier and described more transparently.

As the reviewer notes, these eight algorithms are well established in medical applications; accordingly, we focus the revised description on how they were implemented and evaluated in our study rather than on general algorithm descriptions.

We restructured the Methods section so that the study design, cohort definition, outcome, and predictor time window are presented first, followed immediately by a dedicated machine-learning subsection. This subsection now describes the eight candidate algorithms, the feature-selection workflow, handling of class imbalance, hyperparameter optimization, internal validation, external validation, model-selection criteria, and performance measures. The relation between model development, recalibration, PSI analysis, and SHAP interpretation is also stated explicitly.

To improve concision without reducing reproducibility, routine implementation details were condensed in the main text. Full structured specifications of the preprocessing workflow, hyperparameter search spaces, random seeds, missing-data handling, and class-imbalance strategy are now provided in the relevant supplementary tables (S2-S4).

These changes make the study easier to follow for readers interested in the modelling framework, while preserving the methodological detail required to evaluate validity and reproducibility.

**Location: Methods, Sections 2.2–2.4, Page 8, Line 201 to Page 11, Line 307; Supplementary Tables S3–S4.**

**Comment 4. Results and figures**

*The Results narrative is difficult to follow, and the figures are too blurry to interpret, particularly the SHAP analyses. The predictive performance of the eight machine-learning models appears average compared with similar MIMIC-based studies. The authors should analyse the factors contributing to these results and discuss potential methodological approaches for improvement, rather than repeating tabulated results in the text.*

**Response:**

We appreciate this constructive comment. We agree that the original Results section placed insufficient emphasis on interpretation and that the readability of several figures, particularly the SHAP-based visualizations, was inadequate.

We prepared separately named, publication-ready TIFF working files after reviewing the image dimensions, resolution, and SHAP-panel legibility at final publication size. The SHAP feature-importance, beeswarm, decision, and waterfall plots are retained as clearly labelled panels, and the legends were revised to define each panel and enable interpretation without reliance on the main text.

We reorganized the Results section to reduce repetition of tabulated metrics and to focus on their interpretation. Tree-based models showed better overall discrimination than the linear and distance-based alternatives, consistent with their capacity to accommodate nonlinear associations and interactions among routinely collected ICU variables. We also contextualize the lower local-cohort performance using the observed cross-cohort distributional shift rather than presenting discrimination estimates in isolation.

We have moderated the framing of model performance. The study does not claim state-of-the-art discrimination in a single dataset; rather, it evaluates whether a parsimonious and interpretable model retains useful performance across independent cohorts with differing case mix. We now acknowledge that the restricted predictor set, retrospective data quality, outcome heterogeneity, and distributional differences may limit performance. Prospective multicentre development, richer longitudinal predictors, and local model updating or recalibration are discussed as future directions, not as completed analyses.

Finally, the revised SHAP results explicitly report the main contributors and their direction of association. In MIMIC-III, higher prothrombin time and lactate, and invasive ventilation, generally contributed to higher predicted mortality risk, whereas higher urine output generally contributed to lower predicted risk. These findings make the clinical interpretation that was insufficiently visible in the original figures explicit.

**Location: Results, Sections 3.3 and 3.5, Page 18, Line 493 to Page 22, Line 599; Figures 3–4; Supplementary Figures S1–S3.**

**Comment 5. Discussion and Conclusion**

*The authors did not explain a meaningful contribution beyond already well-known machine-learning implementations in medical prediction. Since the SHAP figures were not visible, it is not possible to evaluate the clinical contribution of this manuscript.*

**Response:**

We appreciate this important comment. We agree that the original Discussion and Conclusion did not sufficiently distinguish the contribution of this study from the routine application of established machine-learning algorithms.

We have revised the manuscript to clarify that the contribution is not the novelty of the individual algorithms. Rather, the study evaluates a parsimonious eight-variable prediction framework for SRMI across two independent external cohorts and examines several complementary dimensions of transportability: discrimination, probability calibration, distributional shift, and model interpretation. This framework allows performance differences across settings to be interpreted in relation to both case-mix heterogeneity and probability miscalibration.

We have also strengthened the clinical interpretation of the SHAP findings. The revised Results and Discussion explicitly report that prothrombin time, invasive ventilation, lactate, temperature, and urine output were the principal contributors to model predictions in the MIMIC-III cohort. Their directions of association are described in clinically interpretable terms: higher prothrombin time and lactate, and invasive ventilation, generally increased predicted mortality risk, whereas higher urine output generally lowered it. We emphasize that these SHAP findings describe how the model used observed predictors and do not establish causal effects.

Finally, we have moderated the Conclusion. The model is presented as an interpretable, externally evaluated approach to early SRMI risk stratification, not as a universally superior or deployment-ready tool. Prospective multicentre validation and local model updating remain necessary before clinical use.

**Location: Discussion, Page 23, Line 639 to Page 27, Line 751; Conclusion, Page 27, Lines 755–767.**

**Comments from Reviewer #2:**

**Comment 1. Predictor extraction and potential temporal leakage**

*The manuscript states that variables were extracted from the first 24 hours after ICU admission. However, it is unclear whether continuous variables represent first recorded values, worst values, averages, maximum abnormalities, or another summary measure. This is particularly important for variables such as invasive ventilation, urine output, lactate, and SOFA score. The authors should clearly define the extraction strategy for every predictor and discuss the possibility of temporal leakage.*

**Response:**

We appreciate the reviewer’s focus on this issue. We agree that the original manuscript did not define the first-24-hour aggregation rules with sufficient precision.

All candidate predictors were restricted to observations recorded from ICU admission until 24 hours after ICU admission. The revised Methods and Supplementary Table S2 now specify the summary rule for every variable. Briefly, SOFA was represented by the first-24-hour derived score; lactate and laboratory variables summarized as severity markers used the maximum recorded value; heart rate, mean blood pressure, respiratory rate, and temperature used the mean recorded value; urine output was the cumulative first-24-hour output; and invasive ventilation and vasopressor use were coded as any exposure during the first 24 hours. Demographic and chronic-comorbidity variables were defined at admission or from pre-existing diagnoses.

We have also clarified the prediction time point and its implications. The model is intended for risk stratification after completion of the first ICU day, not at the instant of ICU admission. Accordingly, no measurements or treatments recorded after the 24-hour predictor window were used for model development or validation. Nevertheless, variables such as invasive ventilation can reflect both disease severity and early clinical management; their inclusion should therefore be interpreted as prediction using information available within the defined clinical window, rather than as evidence of a causal effect. This timing restriction reduces the risk of outcome leakage but does not remove the need for prospective evaluation of a real-time deployment workflow.

**Location: Methods, Section 2.2, Page 8, Lines 209–223; Supplementary Table S2.**

**Comment 2. Clarification of recalibration methodology**

*The recalibration procedure requires additional explanation. The manuscript indicates that calibration parameters were estimated in the development cohort and then applied directly to external cohorts. This differs from conventional external recalibration approaches and may be confusing to readers. The authors should provide a clearer description of the methodology, including how the calibration intercept and slope were estimated and why this approach was chosen.*

**Response:**

We appreciate this important comment. We agree that the original description did not adequately distinguish our transportability-oriented recalibration procedure from conventional cohort-specific external recalibration.

We have clarified that the recalibration mapping was fitted in the development cohort using out-of-fold predicted probabilities. Specifically, repeated stratified cross-validation was used to generate out-of-fold random-forest probabilities for all development-cohort participants. A logistic calibration model was then fitted as logit(P_adj) = alpha + beta × logit(P_pred), where P_pred is the original predicted probability. The fitted intercept (alpha) and slope (beta) defined a fixed mapping that was applied without refitting to the MIMIC-III and local cohorts.

This procedure was not intended to estimate the best calibration model separately in each external cohort. Instead, it assessed whether a development-derived probability-adjustment rule could improve probability alignment when transported unchanged to new settings. We explicitly describe this as a transportability sensitivity analysis and report calibration intercept, calibration slope, and Brier score before and after application in each cohort. Cohort-specific recalibration would require outcome data from the intended deployment population and is described as a separate implementation option rather than as an analysis performed here.

**Location: Methods, Section 2.5, Page 11, Line 309 to Page 12, Line 339; Results, Section 3.4, Page 19, Line 530 to Page 20, Line 558; Supplementary Table S6.**

**Comment 3. Reproducibility and TRIPOD-AI reporting**

*Although the manuscript states that it follows TRIPOD-AI recommendations, several important implementation details are missing or insufficiently described: exact hyperparameter search spaces for each model; random seed settings; details of class-imbalance handling; whether feature selection was performed within each cross-validation fold; full preprocessing workflow reproducibility; and missingness percentages for individual variables.*

**Response:**

We appreciate the reviewer’s identification of these reporting gaps. We agree that the original manuscript did not provide a sufficiently structured implementation specification for reproducibility assessment.

We have provided Supplementary Table S3, which specifies the hyperparameter search space, search strategy, scoring metric, number of sampled parameter sets, cross-validation configuration, random seed, and class-imbalance approach for each of the eight algorithms in the final comparison. Missing values were addressed by k-nearest-neighbours imputation (k = 10) before model development. As reported in Supplementary Table S4, variable-level missingness was below the prespecified 30% exclusion threshold; KNN imputation was used to retain multivariable structure while handling the remaining missing values. Supplementary Table S2 provides the corresponding units, first-24-hour aggregation rules, and source/operational definitions.

We have expanded the main Methods to state the reproducibility settings explicitly, including the random seed (42), repeated stratified 5-fold cross-validation with three repetitions for internal evaluation, stratified 5-fold cross-validation for hyperparameter search, development-derived winsorization thresholds, scaling and encoding, and the imputation and class-weighting procedures used for each model.

Regarding feature selection, we have clarified the current implementation transparently. The RFA-SEIFE procedure used repeated stratified cross-validation to evaluate candidate subsets within the development cohort; however, the final eight-variable set was selected on the full development cohort and then fixed for subsequent model-performance evaluation. Feature selection was therefore not rerun within each outer performance-validation fold. We state this limitation explicitly and do not imply a fully nested feature-selection design. The external cohorts were not used for feature selection or hyperparameter tuning.

Together, these additions align the reporting more closely with TRIPOD-AI principles while accurately reflecting the implemented analysis rather than retrospectively overstating its degree of nesting.

**Location: Methods, Sections 2.3–2.4, Page 9, Line 230 to Page 11, Line 307; Supplementary Tables S3–S4.**

**Comment 4. Missing data handling**

*The authors used KNN imputation with k = 10. Additional justification for this choice is needed. A summary of missingness patterns should be provided, and the potential influence of imputation under cross-cohort distribution shift should be discussed.*

**Response:**

We appreciate the request to justify the imputation strategy and clarify its implications for transportability. We agree that this information is essential for interpreting a model evaluated across heterogeneous cohorts.

We have provided Supplementary Table S4, which reports variable-level pre-imputation missingness for all 31 candidate predictors in the development and external cohorts. All retained predictors were below the prespecified 30% exclusion threshold. The largest missingness percentages were 21.86% for lactate in the development cohort and 23.55% for pH in the MIMIC-III cohort; the local cohort had no missing values for these predictors.

We have clarified that KNN imputation with k = 10 was used before model development to retain the multivariable relationships among the remaining predictors rather than to discard observations with otherwise usable information. We have further clarified in the Discussion that imputation cannot eliminate between-cohort distributional differences and may influence transported probability estimates. Accordingly, the PSI, discrimination, and calibration findings are interpreted as properties of the complete preprocessing-and-modeling workflow, and this potential contribution of imputation to cross-cohort heterogeneity is acknowledged as a limitation.

**Location: Methods, Section 2.3, Page 9, Lines 231–236; Section 2.4, Page 10, Lines 272–279; Discussion, Page 26, Line 727 to Page 27, Line 748; Supplementary Table S4.**

**Comment 5. Comparison with established clinical scores**

*The manuscript discusses SOFA and APACHE II as existing prognostic tools, but no direct performance comparison is presented. Including comparisons against established clinical scoring systems would strengthen the argument for clinical utility and help quantify the added value of the proposed model.*

**Response:**

We appreciate this helpful suggestion. We agree that the clinical value of the model should be assessed against a routinely available severity-score benchmark rather than inferred from standalone performance.

We have expanded the direct comparison between the selected random forest model and SOFA in the development, MIMIC-III external-validation, and local external-validation cohorts (Supplementary Table S5). The paired AUC increment for random forest was 0.181 (95% CI, 0.147 to 0.216; DeLong P < 0.001) in development, 0.082 (95% CI, 0.053 to 0.109; P < 0.001) in MIMIC-III, and 0.098 (95% CI, 0.012 to 0.182; P = 0.022) in the local cohort. We also report stratified-bootstrap confidence intervals (2,000 replicates) for continuous NRI and IDI. Compared with SOFA, both measures were positive in development and MIMIC-III (all bootstrap P < 0.001). In the local cohort, IDI remained significant (0.095; 95% CI, 0.034 to 0.153; P = 0.004), whereas continuous NRI did not reach statistical significance (0.227; 95% CI, −0.055 to 0.494; P = 0.119). We report this latter finding transparently rather than inferring uniform reclassification improvement. The Methods now specify the paired DeLong test, fixed seed, stratified bootstrap procedure, and development-fitted SOFA probability mapping.

APACHE II could not be evaluated reproducibly across all three analytic cohorts because neither a harmonized APACHE II score nor the complete component data required to reconstruct it was available throughout. We state this limitation explicitly and do not claim superiority over APACHE II. The Discussion has been revised to interpret the results as evidence of performance beyond SOFA, while retaining the need for prospective impact evaluation.

**Location: Methods, Section 2.6, Page 13, Lines 341–355; Results, Section 3.3, Page 19, Lines 517–528; Supplementary Table S5.**

**Comment 6. Feature selection procedure**

*The manuscript should explicitly clarify whether feature selection was nested within the cross-validation framework. If feature selection was performed before cross-validation, performance estimates may be optimistically biased.*

**Response:**

We appreciate the reviewer’s emphasis on the risk of optimistic performance estimation. This issue overlaps with Comment 3, and we have revised the Methods to describe the implemented feature-selection procedure transparently.

Specifically, RFA-SEIFE used repeated stratified 5-fold cross-validation with three repetitions to evaluate candidate subsets in the development cohort, and preprocessing for each subset-performance evaluation was fitted on the corresponding training partition only. However, the initial variable ranking and the final eight-predictor set were derived using the full development cohort and then held fixed for subsequent model-performance evaluation. Feature selection was therefore not rerun within each outer performance-validation fold.

We now state explicitly that this non-nested design may yield optimistic internal-performance estimates. The external MIMIC-III and local cohorts were not used for feature selection or hyperparameter tuning; their results therefore remain independent external evaluations of the fixed predictor set. We have avoided characterizing the internal estimates as fully nested cross-validation results.

**Location: Methods, Section 2.3, Page 9, Line 250 to Page 10, Line 258.**

**Comment 7. External validation and generalizability**

*The inclusion of a local external cohort is a strength. However, the local cohort is relatively modest in size. The authors should moderate claims regarding robustness and generalizability and discuss this limitation more explicitly.*

**Response:**

We appreciate this important qualification. We agree that the local cohort (n = 280, with 67 in-hospital deaths) provides an independent real-world test, but its size and single-center origin do not establish broad generalizability or deployment readiness.

We have revised the Abstract, Results, Discussion, and Conclusion to describe the findings as external evaluation in two independent cohorts rather than as evidence of robust or universal generalizability. We now report stratified-bootstrap 95% confidence intervals for the random-forest AUC, PR-AUC, and Brier score in both external cohorts (Supplementary Table S5). The local-cohort intervals are wider than those in MIMIC-III, and we avoid interpreting a favorable point estimate as definitive validation.

The Discussion limitations now state that the local cohort's 67 in-hospital deaths, single-center setting, and observed cross-cohort distributional differences limit precision and transportability. We emphasize that prospective multicenter validation and local recalibration or updating are required before clinical deployment.

**Location: Abstract, Page 1, Line 19 to Page 2, Line 46; Results, Page 18, Line 501 to Page 19, Line 510; Discussion, Page 26, Line 727 to Page 27, Line 751; Conclusion, Page 27, Lines 755–767; Supplementary Tables S5–S6.**

**Comment 8. Data availability and reproducibility**

*The manuscript includes a Data Availability Statement, and the use of publicly accessible MIMIC datasets is appreciated. However, the local cohort data are available only upon reasonable request, and the analysis code is not currently deposited in a public repository. The authors should clarify whether de-identified derived datasets, variable definitions, feature extraction procedures, and model development code can be shared through an appropriate repository to enhance reproducibility and support open science practices.*

**Response:**

We appreciate the reviewer’s emphasis on reproducibility and open science. We agree that the original Data Availability Statement did not provide sufficient detail about the materials that can be shared independently of the source databases and the privacy-restricted local cohort.

We have publicly deposited a versioned reproducibility framework at https://github.com/zxinyuan20-hue/srmi-reproducibility-framework and archived version 1.0.0 at Zenodo (https://doi.org/10.5281/zenodo.21786891). The repository contains framework scripts for feature selection, eight-model comparison, recalibration, visualization, and SHAP interpretation; a variable dictionary; a generic cohort-query template; and a synthetic demonstration workflow. It does not contain patient-level data, original source extracts, or institution-specific phenotyping or adjudication logic. We have revised the Data Availability Statement accordingly. MIMIC-III and MIMIC-IV remain accessible through PhysioNet to credentialed users under the applicable data-use agreements. The local cohort remains restricted because of institutional and privacy requirements and may be available from the corresponding authors on reasonable request and with appropriate ethical approval; no patient-level records are deposited in the public repository.

**Location: Declarations, Availability of data and materials, Page 30, Lines 829–839.**

**Minor Comments from Reviewer #2:**

**Minor Comment 1. Language editing**

*Several grammatical and typographical issues remain throughout the manuscript and would benefit from careful language editing.*

**Response:**

We appreciate this helpful observation. We have completed a full language and typographic edit of the revised manuscript, including the Abstract, Methods, Results, Discussion, figure and table legends, supplementary materials, abbreviations, and cross-references. We also completed a final consistency check between the clean and tracked manuscripts after the scientific revisions were finalized.

**Minor Comment 2. Repeated robustness and generalizability claims**

*Some sections contain repetitive statements regarding robustness and generalizability. These claims should be moderated given the retrospective design and single-center local validation cohort.*

**Response:**

We agree. This concern overlaps with Major Comment 7. We have revised the Abstract, Results, Discussion, and Conclusion to describe the local cohort as an independent but imprecise single-center external evaluation, rather than evidence of universal generalizability or deployment readiness. We also consolidated the remaining robustness language and now state consistently that prospective multicenter validation and local recalibration or updating are needed before clinical use.

**Location: Abstract, Page 1, Line 19 to Page 2, Line 46; Results, Page 18, Line 501 to Page 19, Line 510; Discussion, Page 26, Line 727 to Page 27, Line 751; Conclusion, Page 27, Lines 755–767.**

**Minor Comment 3. PSI rationale and harmonization strategy**

*The PSI-based harmonization analysis is interesting but would benefit from additional methodological justification, including the rationale for the selected thresholds and harmonization strategy.*

**Response:**

We appreciate this suggestion. We have expanded the PSI Methods to specify development-cohort decile bins for continuous variables, category-frequency comparisons for categorical variables, and the descriptive interpretation bands of <0.10, 0.10-0.25, and >0.25 for stable, moderate, and substantial shift, respectively. We also clarify that development-anchored harmonization was a sensitivity analysis used to assess whether simple distribution alignment altered discrimination or calibration; it was not used for primary model training or presented as a remedy for transportability. The revised text makes this rationale and the limits of PSI-based alignment explicit.

**Location: Methods, Section 2.8, Page 14, Line 378 to Page 15, Line 414; Results, Section 3.6, Page 22, Line 616 to Page 23, Line 635; Supplementary Figures S4–S5.**

**Minor Comment 4. Figure, supplement, and abbreviation consistency**

*Please ensure all figure labels, supplementary figure references, and abbreviations are consistently defined and formatted.*

**Response:**

We agree. We have completed a cross-document consistency audit of all figure labels, legends, supplementary figure and table citations, abbreviations, units, and capitalization. Corrections were synchronized between the clean manuscript, tracked manuscript, figures, and supplementary files.

**Comments from Reviewer #3:**

**Major Comment 1. Database-specific operationalization of SRMI**

*The diagnosis depends on troponin above the 99th percentile (or a positive hs-cTn) with acute coronary syndrome (ACS) excluded, based on a 2025 consensus. MIMIC-III covers roughly 2001-2012 and MIMIC-IV roughly 2008-2019, when troponin assays and reference limits differed and hs-cTn was not in routine use. Please state exactly how SRMI was operationalized in each database: which assays and cutoffs were used, how the 99th percentile limit was applied, and how ACS and other causes of troponin rise were excluded (ICD codes, chart review?). Without this it is unclear whether the same condition was captured across cohorts.*

**Response:**

We appreciate the reviewer’s identification of this fundamental issue. We agree that a consensus-based clinical definition cannot be assumed to have been applied equivalently across databases with different assay eras unless the database-specific operationalization is fully reported.

We have revised the cohort-definition section and added Supplementary Table S1. The table reports the database item or local assay, unit, qualifying rule, and time window for each cohort, together with the code-based or chart-review exclusion method. MIMIC-IV used row-level reference limits when available and the laboratory abnormal flag otherwise; MIMIC-III used the abnormal flag because a stable row-level upper reference limit was unavailable; and the local cohort used conventional cTnI measured with the Beckman Coulter Access AccuTnI+3 assay, with a threshold of >40 pg/mL.

We distinguish the consensus-informed clinical concept from the cohort-specific, reproducible algorithms. In MIMIC, ACS and competing causes were excluded by admission-level diagnosis and procedure codes; in the local cohort, they were excluded through medical-record review. The revised Methods and limitations state that these approaches do not establish diagnostic equivalence across cohorts, particularly because the MIMIC code-based exclusions are hospitalization-level proxies without reliable procedure-to-troponin temporal ordering.

**Location: Methods, Section 2.1, Page 6, Line 154 to Page 7, Line 198; Discussion, Page 26, Lines 734–740; Supplementary Table S1.**

**Major Comment 2. Cardiac biomarker reporting and predictor eligibility**

*Troponin defines the cohort, yet it does not appear in Table 1 and is not among the candidate or final predictors. Please report troponin (and any other cardiac markers) across cohorts to characterize the population and to show the definition was applied consistently. If troponin could not be used as a variable, explain why.*

**Response:**

We appreciate this important observation. We agree that reporting the phenotyping biomarker is necessary to characterize the study population and to make clear how the cohort definition was applied in each database.

We have therefore added a dedicated cardiac-biomarker section to Table 1. It reports the number and percentage of patients meeting the cohort-specific troponin-elevation criterion and describes the available first-24-h cardiac-marker measurements by cohort. The table note states explicitly that MIMIC-IV Troponin T is reported in ng/mL, MIMIC-III Troponin I in ng/mL, and local conventional cTnI in pg/mL, reflecting different assays and reference-limit conventions. Raw troponin values are consequently presented as assay-specific concentrations as recorded in the source datasets, rather than pooled or interpreted as a common measurement scale. In the local cohort, conventional cTnI, CK-MB, and BNP/NT-proBNP are summarized as their first-24-h maxima.

Troponin was excluded from the candidate predictor set a priori because it was used to define the analytic phenotype. Conditioning enrollment on troponin elevation and then using an assay-dependent troponin value as a predictor would conflate phenotype ascertainment with prognostic modeling and would not be transportable across the three assay environments. This was a design decision rather than an assertion that troponin has no prognostic information. CK-MB and BNP/NT-proBNP were retained for clinical characterization in the local cohort but were not part of the cross-cohort candidate predictor set or the SRMI eligibility rule.

**Location: Methods, Section 2.1, Page 7, Lines 183–198; Table 1.**

**Major Comment 3. Cohort exclusions and missing-data handling**

*Nearly half of otherwise eligible patients were dropped for missing key variables. This is the largest step in cohort selection and likely biases the sample toward sicker patients who receive more testing. The text also states that KNN imputation was used, which seems to conflict with excluding patients for missingness. Please clarify which variables required complete data and why, compare included versus excluded patients, and justify the approach.*

**Response:**

We appreciate the reviewer’s identification of this important ambiguity. We agree that the original flowchart label, "missing key variables," did not explain the missingness screen and could therefore be read as inconsistent with the reported KNN imputation.

We have revised Figure 1 to distinguish the sequential eligibility steps. The initial SRMI cohort was defined using Sepsis-3, a qualifying troponin elevation, and diagnosis-based exclusion of acute competing conditions (including acute coronary syndrome and cardiac arrest, as appropriate to the cohort-specific operationalization). Procedure- or injury-related competing causes of myocardial injury, including cardiac surgery or intervention, cardiopulmonary resuscitation, and severe chest trauma, were then excluded as a separate step. These exclusions are distinct from missing-data handling.

The former label "missing key variables" has been replaced with "overall missingness across candidate predictors >30%." Patients were excluded at this stage when the proportion of missing values across their candidate-predictor profile exceeded the prespecified 30% threshold; no individual candidate predictor was required to be complete. Among patients retained after this screen, residual missing values were imputed using k-nearest neighbours (k = 10). We have made this sequence explicit in the Methods and Figure 1 and cite Supplementary Table S4 for predictor-specific pre-imputation missingness.

Because this patient-level screen excludes patients precisely for substantial unavailability of candidate predictors, a comprehensive included-versus-excluded comparison cannot be made using information that is comparable across groups. Rather than draw potentially misleading contrasts from selectively observed variables, we report all flowchart denominators and have acknowledged in the Discussion that the >30% missingness screen may introduce selection bias. KNN imputation among retained patients does not remove this potential bias.

**Location: Methods, Section 2.3, Page 9, Lines 231–233; Figure 1; Discussion, Page 26, Line 727 to Page 27, Line 748; Supplementary Table S4.**

**Major Comment 4. Recalibration coefficients and development-cohort calibration**

*The authors state that the calibration intercept and slope were estimated only in the development cohort and applied to the external cohorts without refitting. A logistic recalibration fitted on the same data used to train the model should give an intercept near 0 and a slope near 1, which would barely change external calibration. Yet the MIMIC-III intercept moves from -0.635 to -0.188 and the slope from 1.249 to 1.081, which requires non-trivial coefficients. Please report the actual recalibration coefficients and the development-cohort calibration (before and after), and explain what systematic effect the fixed mapping is correcting (for example, probability distortion introduced by class weighting). As written, the mechanism is unclear and the numbers do not follow obviously from the stated method.*

**Response:**

We appreciate this important observation. The key clarification is that the logistic recalibration mapping was not fitted to resubstitution predictions from the final random forest. It was fitted to repeated stratified 5-fold out-of-fold (OOF) probabilities in the development cohort (three repetitions; n = 1,226), and the resulting fixed mapping was subsequently applied to probabilities from the final development model and to both external cohorts. Thus, the recalibration fit was based on held-out development predictions, not on the same predictions used to train the final random forest.

The OOF-fitted mapping was logit(P_adj) = -0.414 + 1.156 × logit(P_pred). We have reported these coefficients explicitly in the Methods and Supplementary Table S6. When this mapping was applied to the final development-model probabilities, the calibration intercept, slope, and Brier score changed from -0.452, 2.301, and 0.118 to 0.372, 1.992, and 0.109, respectively. These values are not expected to be exactly 0 and 1 because the mapping was fitted to OOF probabilities whereas the reported development-model assessment used predictions from the model refitted on the full development cohort.

For completeness, the same fixed mapping changed the MIMIC-III calibration intercept, slope, and Brier score from -0.635, 1.249, and 0.162 to -0.188, 1.081, and 0.147, respectively; in the local cohort, the corresponding values changed from -1.498, 1.093, and 0.233 to -1.107, 0.946, and 0.196. As expected for a monotonic logistic transformation, discrimination was unchanged.

We have also clarified the interpretation of this result. The fixed mapping partially corrected a systematic probability-scale mismatch that was visible in the OOF development predictions and transported to the external cohorts. This is compatible with, but does not prove, an influence of class weighting together with the probability behavior of the random-forest ensemble. We therefore avoid attributing the effect uniquely to class weighting and describe recalibration as a fixed, development-derived probability adjustment rather than a replacement for local recalibration or updating.

**Location: Methods, Section 2.5, Page 12, Lines 314–339; Results, Section 3.4, Page 19, Line 530 to Page 20, Line 558; Supplementary Table S6.**

**Major Comment 5. Interpretation of calibration intercept and slope**

*A slope above 1 (1.249) usually means predictions are too moderate (under-dispersed), not "over-dispersion." A negative intercept usually means overestimation, not underestimation. Overestimation is also what one would expect here, since mortality is lower in the external cohorts (29% and 24%) than in development (35%). Please re-check both interpretations.*

**Response:**

We appreciate the reviewer’s identification of this error in our calibration terminology. We agree that the previous interpretation was incorrect and have revised the Results and Discussion accordingly.

Using the standard logistic calibration model of observed outcome on logit(predicted probability), a slope above 1 indicates under-dispersion: the predicted probabilities are too moderate and do not vary sufficiently across the observed risk range. A negative calibration intercept indicates systematic risk overestimation. We have therefore replaced the previous descriptions of "over-dispersion" and "underestimation" in the MIMIC-III results.

Specifically, before recalibration in MIMIC-III, the intercept of -0.635 and slope of 1.249 are described as risk overestimation and under-dispersion. The corresponding local-cohort values (-1.498 and 1.093) are interpreted in the same direction. The fixed development-derived mapping improved these metrics toward zero and one, respectively, but we make clear that this partial improvement does not establish optimal local calibration or remove the need for local assessment and updating before clinical use. This interpretation is also consistent with the lower event rates in the external cohorts (29.2% in MIMIC-III and 23.9% in the local cohort) than in the development cohort (34.5%): a model fitted in a higher-risk development population would be expected to overestimate risk when applied to lower-risk external populations.

**Location: Results, Section 3.4, Page 20, Lines 539–550; Discussion, Page 25, Lines 685–695.**

**Major Comment 6. Benchmark comparison with SOFA**

*SOFA and APACHE II are described as inadequate, but the model is never compared against SOFA (which is one of its own inputs) or any baseline score. The central clinical question is whether the model adds value beyond a routine severity score. Please add this comparison, at least against SOFA, in all three cohorts.*

**Response:**

We agree that a direct benchmark against a routine severity score is essential. We have therefore compared the final random forest model with SOFA in the development, MIMIC-III, and local cohorts and report discrimination, precision-recall performance, Brier score, paired AUC differences, continuous net reclassification improvement (NRI), and integrated discrimination improvement (IDI) in Supplementary Table S5.

The random forest showed higher AUC than SOFA in all cohorts: 0.810 versus 0.628 in development, 0.819 versus 0.737 in MIMIC-III, and 0.758 versus 0.660 in the local cohort. The paired AUC increments were 0.181 (95% CI, 0.147-0.216; P < 0.001), 0.082 (0.053-0.109; P < 0.001), and 0.098 (0.012-0.182; P = 0.022), respectively. NRI and IDI were positive in all cohorts; in the local cohort, the NRI was imprecise and not statistically significant, whereas IDI remained positive.

Because SOFA is one of the candidate predictors, this comparison evaluates the incremental prognostic information supplied by the multivariable model beyond a routinely available severity score; it does not imply independence from SOFA. APACHE II was not compared because no harmonized APACHE II score or complete component data were available across all three cohorts. We have revised the Methods, Results, and Discussion to present this as a severity-score benchmark rather than a claim of clinical benefit or replacement of established scores.

**Location: Methods, Section 2.6, Page 13, Lines 341–355; Results, Section 3.3, Page 19, Lines 517–528; Supplementary Table S5.**

**Major Comment 7. Confidence intervals for external performance**

*External AUCs are given as single point estimates. Please report 95% confidence intervals for AUC, PR-AUC, Brier score, and the calibration metrics in both external cohorts. This is especially important for the local cohort.*

**Response:**

We agree. We have added percentile bootstrap 95% confidence intervals based on 2,000 stratified nonparametric replicates for AUC, PR-AUC, Brier score, calibration intercept, and calibration slope in both external cohorts (Supplementary Tables S5 and S6).

In MIMIC-III, the random forest AUC was 0.819 (95% CI, 0.787-0.848), PR-AUC 0.702 (0.657-0.751), and Brier score 0.147 (0.136-0.158). In the local cohort, the corresponding estimates were 0.758 (0.693-0.816), 0.525 (0.435-0.630), and 0.196 (0.176-0.217). We have revised the Abstract and Results to report these intervals and have added interval estimates for the calibration metrics in Supplementary Table S6.

**Location: Abstract, Page 2, Lines 34–46; Results, Section 3.3, Page 18, Line 501 to Page 19, Line 510; Supplementary Tables S5–S6.**

**Major Comment 8. Precision of local-cohort external validation**

*With 67 deaths, the local validation is below the commonly recommended minimum (about 100 events and 100 non-events) for reliable external validation, particularly for calibration. Please acknowledge this and soften the conclusions about local performance.*

**Response:**

We agree that the local cohort, with 67 in-hospital deaths, provides an independent but imprecise external evaluation. We have revised the Abstract, Results, Discussion, and Conclusion to avoid interpreting this cohort as evidence of broad generalizability, clinical deployment readiness, or precise local calibration.

The wider local bootstrap intervals are now reported explicitly, and the Discussion notes that the single-center cohort and limited event count constrain precision, especially for calibration assessment. We describe the local results as a setting-specific external test of a fixed predictor set and emphasize that prospective multicenter validation, local evaluation, and recalibration or updating are required before clinical use.

**Location: Abstract, Page 1, Line 19 to Page 2, Line 46; Results, Page 18, Line 501 to Page 19, Line 510; Discussion, Page 26, Line 727 to Page 27, Line 751; Conclusion, Page 27, Lines 755–767.**

**Major Comment 9. Class weighting and probability calibration**

*Class weighting was applied to several models including random forest. This distorts predicted probabilities and is a likely source of the miscalibration that recalibration later corrects. Please discuss this trade-off and consider whether a model without weighting (using threshold tuning instead) would produce better-calibrated probabilities.*

**Response:**

We appreciate the reviewer’s focus on this point. We agree that class weighting is a discrimination-oriented strategy and can affect the scale of predicted probabilities. We have revised the Methods and Discussion to state this trade-off explicitly. The observed mismatch cannot be attributed uniquely to class weighting because random-forest probability behavior, model specification, and cohort shift may also contribute; recalibration is therefore not presented as proof of a single mechanism.

We conducted an unweighted-random-forest sensitivity analysis using the same eight predictors, fixed hyperparameters, development-derived preprocessing, and external cohorts as in the primary analysis. In MIMIC-III, unweighted versus weighted random forest had AUC 0.820 versus 0.819 (difference, 0.002; 95% CI, -0.002 to 0.005), PR-AUC 0.705 versus 0.702 (0.003; -0.004 to 0.009), and Brier score 0.148 versus 0.162 (-0.014; -0.016 to -0.011). In the local cohort, the corresponding values were 0.755 versus 0.758 (-0.003; -0.012 to 0.006), 0.523 versus 0.525 (-0.002; -0.019 to 0.015), and 0.191 versus 0.233 (-0.041; -0.047 to -0.036). Calibration intercepts moved toward zero in both external cohorts, while slopes remained above 1.

Probability metrics were assessed before threshold selection. For threshold-dependent descriptive measures, thresholds were selected from repeated development-cohort out-of-fold probabilities by the Youden index (0.295 for unweighted and 0.384 for weighted random forest) and transported unchanged to external cohorts; balanced accuracy remained similar. The full comparison is reported in Supplementary Table S7 and summarized in the Results. We interpret it as evidence that class weighting can contribute to probability-scale distortion, not as evidence that either strategy is universally preferable or that local recalibration is unnecessary.

The weighted random forest was retained as the primary model because it was selected by the prespecified primary model-comparison workflow, whereas the unweighted model was a post hoc sensitivity analysis rather than a model re-selection exercise. After the fixed development-derived recalibration, the MIMIC-III Brier score of the weighted model was 0.147, comparable to 0.148 for the unweighted model before recalibration. We therefore interpret the sensitivity analysis as identifying a probability-calibration consideration, not as establishing that the unweighted model should replace the prespecified primary model.

**Location: Methods, Section 2.4, Page 10, Line 280 to Page 11, Line 295; Results, Section 3.6, Page 22, Lines 602–615; Discussion, Page 25, Line 696 to Page 26, Line 712; Supplementary Table S7.**

**Major Comment 10. Consistency of reported SHAP predictors**

*The abstract and SHAP results highlight prothrombin time and invasive ventilation as the top predictors, but the Discussion lists ‘SOFA score, lactate, age, and urine output’ as the key contributors. These do not agree. Please make the leading predictors consistent throughout.*

**Response:**

We appreciate the reviewer’s identification of this inconsistency. We agree that the manuscript should distinguish the selected predictor set from the predictors with the largest SHAP contributions. The eight variables were retained in the final model, whereas the SHAP ranking in the MIMIC-III validation cohort identified prothrombin time, invasive ventilation, lactate, temperature, and urine output as the leading contributors to model output.

We have revised the Abstract, Results, Discussion, and relevant figure text to use this same ordered set when describing the principal SHAP contributors. SOFA score, age, and respiratory rate are described as final-model predictors rather than as the leading SHAP contributors unless a specific result supports that claim. We also make clear that the ranking is based on mean absolute SHAP values in the MIMIC-III cohort and is descriptive of model behavior, not causal importance.

**Location: Abstract, Page 2, Lines 41–46; Results, Section 3.5, Page 20, Line 560 to Page 22, Line 599; Discussion, Page 24, Line 677 to Page 25, Line 684; Figure 4; Supplementary Figures S1–S3.**
