# Supplementary material for: A machine learning model for in-hospital mortality prediction in patients with sepsis-related myocardial injury
Source: PLOS Digit Health. 2026 Oct 12;5(10):e0001758. doi: 10.1371/journal.pdig.0001758 (PMC13657301; doi:10.1371/journal.pdig.0001758)
Supplement: Peer review history

# Peer review: Decision Letter 0

**DOI:** 10.1371/journal.pdig.0001758.r001

**Authors:** Henry Horng-Shing Lu

10 Jul 2026

Response to Reviewers Revised Manuscript with Track Changes Manuscript **Journal Requirements:**

1. Please amend your detailed Financial Disclosure statement. This is published with the article. It must therefore be completed in full sentences and contain the exact wording you wish to be published.

i. Please clarify all sources of financial support for your study. List the grants, grant numbers, and organizations that funded your study, including funding received from your institution. Please note that suppliers of material support, including research materials, should be recognized in the Acknowledgements section rather than in the Financial Disclosure.

ii. State the initials, alongside each funding source, of each author to receive each grant. For example: "This work was supported by the National Institutes of Health (####### to AM; ###### to CJ) and the National Science Foundation (###### to AM)."

iii. State what role the funders took in the study. If the funders had no role in your study, please state: “The funders had no role in study design, data collection and analysis, decision to publish, or preparation of the manuscript.”

iv. If any authors received a salary from any of your funders, please state which authors and which funders.

2. In the online submission form, you indicated that “The minimal data set is available from the corresponding author”.

All PLOS journals now require all data underlying the findings described in their manuscript to be freely available to other researchers, either

1. In a public repository,

2. Within the manuscript itself, or

3. Uploaded as supplementary information.

This policy applies to all data except where public deposition would breach compliance with the protocol approved by your research ethics board. If your data cannot be made publicly available for ethical or legal reasons (e.g., public availability would compromise patient privacy), please explain your reasons by return email and your exemption request will be escalated to the editor for approval. Your exemption request will be handled independently and will not hold up the peer review process, but will need to be resolved should your manuscript be accepted for publication. One of the Editorial team will then be in touch if there are any issues.

3. Please ensure that your Ethics Statement is available in its entirety at the beginning of your Methods section, under a subheading 'Ethics Statement'.

4. Please provide separate figure files in .tif or .eps format.

For more information about figure files please see our guidelines:

https://journals.plos.org/digitalhealth/s/figures

https://journals.plos.org/digitalhealth/s/figures#loc-file-requirements

5. Please ensure that the Title in your manuscript file and the Title provided in your online submission form are the same.

6. Please provide an Author Summary. This should appear in your manuscript between the Abstract (if applicable) and the Introduction, and should be 150–200 words long. The aim should be to make your findings accessible to a wide audience that includes both scientists and non-scientists. Sample summaries can be found on our website under Submission Guidelines:

https://journals.plos.org/digitalhealth/s/submission-guidelines#loc-parts-of-a-submission

**Additional Editor Comments (if provided):** **Reviewers' Comments:**

**Comments to the Author**

1. Does this manuscript meet PLOS Digital Health’s publication criteria?

Reviewer #1: Partly

Reviewer #2: Yes

Reviewer #3: Partly

**********

2. Has the statistical analysis been performed appropriately and rigorously?

Reviewer #1: Yes

Reviewer #2: Yes

Reviewer #3: No

**********

3. Have the authors made all data underlying the findings in their manuscript fully available (please refer to the Data Availability Statement at the start of the manuscript PDF file)?

The PLOS Data policy

Reviewer #1: No

Reviewer #2: No

Reviewer #3: No

**********

4. Is the manuscript presented in an intelligible fashion and written in standard English?

Reviewer #1: Yes

Reviewer #2: Yes

Reviewer #3: Yes

**********

**Reviewer #1:**  The authors of the manuscript titled "Machine learning model for in-hospital mortality prediction in patients with sepsis-related myocardial injury.” utilized machine learning (ML) methods to predict mortality of patients with sepsis related myocardial injury. They implemented eight classification algorithms, including logistic regression, random forest, extreme gradient boosting (XGBoost), extra trees, support vector machine (SVM), k-nearest neighbors (KNN), multilayer perceptron (MLP), and AdaBoost. They also applied PSI and SHAP analyses. The topic of the manuscript is relevant. Comments:

1. The abstract doesn’t support the title of the manuscript. It’s too long and doesn’t describe the methodology nor present results effectively. The authors only mentioned the random forest model in the abstract's methodology section. The results are not well presented; it’s difficult to understand how good the machine learning methods were. Only random forests are mentioned, and it’s not clear what SHAP analysis contributes in this work. This abstract doesn’t accurately reflect the manuscript's text and doesn’t support the manuscript's title. It has to be improved significantly. What are the authors trying to improve with this research and why?

2. The introduction of the manuscript is well written.

3. Methods section. This section is too long, with many unnecessary details. The topic of the manuscript is ML for in-hospital prediction. ML methods are introduced too late in the methods; readers lose interest in this article before they even start reading about ML. The general impression is that this section is too long with a lot of descriptions that are not important for the study. On the other side, the most important element of the research, ML methods, are hardly described. The authors selected 8 basic ML methods that are well described in medical applications.

4. Results. The authors provide a narrative that is not easy to follow. The figures are impossible to read, they are too blurry. It’s impossible to see what the findings of the SHAP analysis are. Predictive results of the 8 ML models are average. Many published studies using MIMIC data achieved better results in similar applications. The authors need to analyze what contributed to these prediction results and how they can be improved. Instead of repeating results in the text of this section, which are already presented in tables, the authors should provide an analysis of the results. Parts of this section belong in the methodology section.

5. Discussion and Conclusion. The authors didn’t explain any meaningful contribution beyond already well-known ML implementations in medical predictions. Since SHAP figures are not visible, it’s not possible to talk about the clinical contribution of this manuscript.

Overall, the contribution of this manuscript is not recognizable. The authors should spend more time and space describing the ML models and explaining why their results are average comparing to results in already published similar studies. Are any methodological solutions available to improve prediction results?

**Reviewer #2:**  This manuscript presents a machine learning model for predicting in-hospital mortality in patients with sepsis-related myocardial injury (SRMI) using a development cohort from MIMIC-IV and external validation cohorts from MIMIC-III and a local clinical dataset. The study addresses an important clinical problem and includes several strengths, notably external validation across multiple cohorts, calibration assessment, dataset-shift analysis using the Population Stability Index (PSI), and SHAP-based interpretability. The manuscript is generally well organized and the topic is relevant to the scope of the journal.

However, several methodological and reporting issues require clarification before the work can be adequately evaluated and considered for publication.

Major Comments

1. Predictor extraction and potential temporal leakage

The manuscript states that variables were extracted from the first 24 hours after ICU admission. However, it is unclear whether continuous variables represent first recorded values, worst values, averages, maximum abnormalities, or another summary measure. This is particularly important for variables such as invasive ventilation, urine output, lactate, and SOFA score. The authors should clearly define the extraction strategy for every predictor and discuss the possibility of temporal leakage.

2. Clarification of recalibration methodology

The recalibration procedure requires additional explanation. The manuscript indicates that calibration parameters were estimated in the development cohort and then applied directly to external cohorts. This differs from conventional external recalibration approaches and may be confusing to readers. The authors should provide a clearer description of the methodology, including how the calibration intercept and slope were estimated and why this approach was chosen.

3. Reproducibility and TRIPOD-AI reporting

Although the manuscript states that it follows TRIPOD-AI recommendations, several important implementation details are missing or insufficiently described:

* Exact hyperparameter search spaces for each model.

* Random seed settings.

* Details of class imbalance handling for all algorithms.

* Whether feature selection was performed within each cross-validation fold.

* Full preprocessing workflow reproducibility.

* Missingness percentages for individual variables.

Additional reporting would improve transparency and reproducibility.

4. Missing data handling

The authors used KNN imputation with k = 10. Additional justification for this choice is needed. A summary of missingness patterns should be provided, and the potential influence of imputation under cross-cohort distribution shift should be discussed.

5. Comparison with established clinical scores

The manuscript discusses SOFA and APACHE II as existing prognostic tools, but no direct performance comparison is presented. Including comparisons against established clinical scoring systems would strengthen the argument for clinical utility and help quantify the added value of the proposed model.

6. Feature selection procedure

The manuscript should explicitly clarify whether feature selection was nested within the cross-validation framework. If feature selection was performed before cross-validation, performance estimates may be optimistically biased.

7. External validation and generalizability

The inclusion of a local external cohort is a strength. However, the local cohort is relatively modest in size. The authors should moderate claims regarding robustness and generalizability and discuss this limitation more explicitly.

8. Data availability and reproducibility

The manuscript includes a Data Availability Statement, and the use of publicly accessible MIMIC datasets is appreciated. However, the local cohort data are available only upon reasonable request, and the analysis code is not currently deposited in a public repository. The authors should clarify whether de-identified derived datasets, variable definitions, feature extraction procedures, and model development code can be shared through an appropriate repository to enhance reproducibility and support open science practices.

Minor Comments

1. Several grammatical and typographical issues remain throughout the manuscript and would benefit from careful language editing.

2. Some sections contain repetitive statements regarding robustness and generalizability. These claims should be moderated given the retrospective design and single-center local validation cohort.

3. The PSI-based harmonization analysis is interesting but would benefit from additional methodological justification, including the rationale for the selected thresholds and harmonization strategy.

4. Please ensure all figure labels, supplementary figure references, and abbreviations are consistently defined and formatted.

Overall Assessment

This study addresses a clinically meaningful problem and includes several methodological strengths, particularly external validation, calibration assessment, and dataset-shift analysis. However, important methodological details remain unclear, particularly regarding predictor extraction, recalibration, feature selection, and reproducibility. Addressing these issues would substantially strengthen the manuscript and improve confidence in the reported findings.

**Reviewer #3:**  This manuscript reports the development and external validation of a random forest model for in-hospital mortality in patients with sepsis-related myocardial injury (SRMI). MIMIC-IV is used as the development cohort, with MIMIC-III and a single-center local cohort for external validation. The authors apply a two-stage feature selection method, logistic recalibration, a population stability index (PSI) analysis of distribution shift, and SHAP for interpretation. An 8-variable model reaches an AUC of 0.819 in MIMIC-III and 0.758 in the local cohort, and recalibration improves the reported calibration metrics.

The question is clinically relevant, and validating in two external cohorts (including real-world local data) is a genuine strength. The attention to calibration and to distribution shift is welcome and is often missing in this literature. That said, several issues need to be addressed before the work can be considered further.

Major comments

1.The diagnosis depends on troponin above the 99th percentile (or a positive hs-cTn) with acute coronary syndrome (ACS) excluded, based on a 2025 consensus. MIMIC-III covers roughly 2001–2012 and MIMIC-IV roughly 2008–2019, when troponin assays and reference limits differed and hs-cTn was not in routine use. Please state exactly how SRMI was operationalized in each database: which assays and cutoffs were used, how the 99th percentile limit was applied, and how ACS and other causes of troponin rise were excluded (ICD codes, chart review?). Without this it is unclear whether the same condition was captured across cohorts.

2.Troponin defines the cohort, yet it does not appear in Table 1 and is not among the candidate or final predictors. Please report troponin (and any other cardiac markers) across cohorts to characterize the population and to show the definition was applied consistently. If troponin could not be used as a variable, explain why.

3.Nearly half of otherwise eligible patients were dropped for missing key variables. This is the largest step in cohort selection and likely biases the sample toward sicker patients who receive more testing. The text also states that KNN imputation was used, which seems to conflict with excluding patients for missingness. Please clarify which variables required complete data and why, compare included versus excluded patients, and justify the approach.

4.The authors state that the calibration intercept and slope were estimated only in the development cohort and applied to the external cohorts without refitting. A logistic recalibration fitted on the same data used to train the model should give an intercept near 0 and a slope near 1, which would barely change external calibration. Yet the MIMIC-III intercept moves from −0.635 to −0.188 and the slope from 1.249 to 1.081, which requires non-trivial coefficients. Please report the actual recalibration coefficients and the development-cohort calibration (before and after), and explain what systematic effect the fixed mapping is correcting (for example, probability distortion introduced by class weighting). As written, the mechanism is unclear and the numbers do not follow obviously from the stated method.

5.A slope above 1 (1.249) usually means predictions are too moderate (under-dispersed), not "over-dispersion." A negative intercept usually means overestimation, not underestimation. Overestimation is also what one would expect here, since mortality is lower in the external cohorts (29% and 24%) than in development (35%). Please re-check both interpretations.

6.SOFA and APACHE II are described as inadequate, but the model is never compared against SOFA (which is one of its own inputs) or any baseline score. The central clinical question is whether the model adds value beyond a routine severity score. Please add this comparison, at least against SOFA, in all three cohorts.

7.External AUCs are given as single point estimates. Please report 95% confidence intervals for AUC, PR-AUC, Brier score, and the calibration metrics in both external cohorts. This is especially important for the local cohort.

8.With 67 deaths, the local validation is below the commonly recommended minimum (about 100 events and 100 non-events) for reliable external validation, particularly for calibration. Please acknowledge this and soften the conclusions about local performance.

9.Class weighting was applied to several models including random forest. This distorts predicted probabilities and is a likely source of the miscalibration that recalibration later corrects. Please discuss this trade-off and consider whether a model without weighting (using threshold tuning instead) would produce better-calibrated probabilities.

10.The abstract and SHAP results highlight prothrombin time and invasive ventilation as the top predictors, but the Discussion lists "SOFA score, lactate, age, and urine output" as the key contributors. These do not agree. Please make the leading predictors consistent throughout.

Minor comments

1.The Methods text and the flowchart (Figure 1) list different exclusion criteria. The text mentions ACS, PCI/thrombolysis, myocarditis, trauma, cardiac surgery, and pregnancy, while the flowchart shows only age, ICU stay, postoperative status, and missing data. Please reconcile.

2.SHAP was run on the MIMIC-III cohort rather than on the development data. This is an unusual choice for explaining a model. Please justify it briefly.

3.The classification metrics (accuracy, F1, recall, precision) depend on the decision threshold, which is not stated. Please report the threshold and how it was chosen. The very high recall and low precision in the local cohort suggest the default threshold may not fit that population.

4.Stating that the minimal dataset and code are "available from the corresponding author on request" may not meet PLOS sharing requirements. Please deposit the analysis code and the minimal dataset, or clearly state the specific legal/ethical restriction for the local data.

**********

what does this mean?). If published, this will include your full peer review and any attached files.

**Do you want your identity to be public for this peer review?** If you choose “no”, your identity will remain anonymous but your review may still be made public.

For information about this choice, including consent withdrawal, please see our Privacy Policy

Reviewer #1: No

Reviewer #2: No

Reviewer #3: No

**********

**Figure resubmission:**

**Reproducibility:**

To enhance the reproducibility of your results, we recommend that authors of applicable studies deposit laboratory protocols in protocols.io, where a protocol can be assigned its own identifier (DOI) such that it can be cited independently in the future. Additionally, PLOS ONE offers an option to publish peer-reviewed clinical study protocols. Read more information on sharing protocols at https://plos.org/protocols?utm_medium=editorial-email&utm_source=authorletters&utm_campaign=protocols

# Author comment: Author response to Decision Letter 1

**DOI:** 10.1371/journal.pdig.0001758.r002

4 Aug 2026

Attachment Submitted filename: Response to Reviewers.docx

# Peer review: Decision Letter 1

**DOI:** 10.1371/journal.pdig.0001758.r003

**Authors:** Henry Horng-Shing Lu

16 Sep 2026

A machine learning model for in-hospital mortality prediction in patients with sepsis-related myocardial injury

PDIG-D-26-00759R1

Dear Dr Yan,

We are pleased to inform you that your manuscript 'A machine learning model for in-hospital mortality prediction in patients with sepsis-related myocardial injury' has been provisionally accepted for publication in PLOS Digital Health.

Before your manuscript can be formally accepted you will need to complete some formatting changes, which you will receive in a follow-up email from a member of our team.

Please note that your manuscript will not be scheduled for publication until you have made the required changes, so a swift response is appreciated.

IMPORTANT: The editorial review process is now complete. PLOS will only permit corrections to spelling, formatting or significant scientific errors from this point onwards. Requests for major changes, or any which affect the scientific understanding of your work, will cause delays to the publication date of your manuscript.

If your institution or institutions have a press office, please notify them about your upcoming paper to help maximize its impact. If they'll be preparing press materials, please inform our press team as soon as possible -- no later than 48 hours after receiving the formal acceptance. Your manuscript will remain under strict press embargo until 2 pm Eastern Time on the date of publication. For more information, please contact digitalhealth@plos.org.

Thank you again for supporting Open Access publishing; we are looking forward to publishing your work in PLOS Digital Health.

Best regards,

Henry Horng-Shing Lu

Section Editor

PLOS Digital Health

***********************************************************

**Additional Editor Comments (if provided):**

**Reviewer Comments (if any, and for reference):**

Reviewer's Responses to Questions

**Comments to the Author**

Reviewer #1: All comments have been addressed

Reviewer #3: All comments have been addressed

**********

publication criteria?

Reviewer #1: Yes

Reviewer #3: Yes

**********

3. Has the statistical analysis been performed appropriately and rigorously?

Reviewer #1: Yes

Reviewer #3: Yes

**********

4. Have the authors made all data underlying the findings in their manuscript fully available (please refer to the Data Availability Statement at the start of the manuscript PDF file)?

The PLOS Data policy

Reviewer #1: Yes

Reviewer #3: Yes

**********

5. Is the manuscript presented in an intelligible fashion and written in standard English?

Reviewer #1: Yes

Reviewer #3: Yes

**********

Reviewer #1: The authors reasonably responded to my comments and provided solid improvements in the text of the manuscript.

Reviewer #3: No other comments.

**********

what does this mean?). If published, this will include your full peer review and any attached files.

**Do you want your identity to be public for this peer review?** If you choose “no”, your identity will remain anonymous but your review may still be made public.

For information about this choice, including consent withdrawal, please see our Privacy Policy

Reviewer #1: No

Reviewer #3: No

**********
